# The developmental factor TBX3 engages with the Wnt/β-catenin transcriptional complex in colorectal cancer to regulate metastasis genes

**DOI:** 10.1073/pnas.2419691122

**Published:** 2025-05-09

**Authors:** Amaia Jauregi-Miguel, Simon Söderholm, Tamina Weiss, Anna Nordin, Valeria Ghezzi, Salome M. Brütsch, Pierfrancesco Pagella, Yorick van de Grift, Gianluca Zambanini, Jacopo Ulisse, Alessandro Mattia, Ruslan Deviatiiarov, Elena Faustini, Lavanya Moparthi, Wenjing Zhong, Bergthor Björnsson, Per Sandström, Erik Lundqvist, Francisca Lottersberger, Stefan Koch, Andreas E. Moor, Xiao-Feng Sun, Eleonore von Castelmur, Guojun Sheng, Claudio Cantù

**Affiliations:** ^a^Wallenberg Centre for Molecular Medicine, Linköping University, Linköping 58183, Sweden; ^b^Department of Biomedical and Clinical Sciences, Division of Molecular Medicine and Virology, Faculty of Medicine and Health Sciences, Linköping University, Linköping 58225, Sweden; ^c^Science for Life Laboratory, SciLifeLab, Linköping University, Linköping 58183, Sweden; ^d^Department of Physics, Chemistry, and Biology, Division of Biophysics and Bioengineering, Faculty of Science and Engineering, Linköping University, Linköping 58183, Sweden; ^e^Regulatory Genomics Research Center, Institute of Fundamental Medicine and Biology, Kazan Federal University, Kazan, Tatarstan 420012, Russian Federation; ^f^Endocrinology Research Center, Moscow 115478, Russian Federation; ^g^Graduate School of Medicine, Juntendo University, Tokyo 113-8421, Japan; ^h^Department of Surgery in Linköping, Linköping University, Linköping 58225, Sweden; ^i^Department of Biomedicine and Clinical Sciences, Linköping University, Linköping 58225, Sweden; ^j^Department of Surgery, Vrinnevi Hospital, Norrköping, Linköping University, Norrköping 60379, Sweden; ^k^Department of Biosystems Science and Engineering, Federal Institute of Technology Zürich, Basel 4056, Switzerland; ^l^Department of Oncology, Division of Surgery, Orthopedics and Oncology, Faculty of Medicine and Health Sciences, Linköping University, Linköping 58225, Sweden; ^m^Department of Biomedical and Clinical Sciences, Division of Surgery, Orthopedics and Oncology, Faculty of Medicine and Health Sciences, Linköping University, Linköping 58225, Sweden; ^n^Department of Physics, Chemistry and Biology, Division of Chemistry, Faculty of Science and Engineering, Linköping University, Linköping 58183, Sweden; ^o^International Research Center for Medical Sciences, Kumamoto University, Kumamoto 860-8555, Japan

**Keywords:** Wnt signaling, β-catenin, TBX3, CUT&RUN, proximity proteomics

## Abstract

Wnt signaling orchestrates gene expression in a plethora of processes during development and adult cell homeostasis via the action of nuclear β-catenin. Yet, little is known about how β-catenin generates context-specific transcriptional outcomes. Understanding this will reveal how aberrant Wnt/β-catenin signaling causes neoplasia specifically of the colorectal epithelium. We have previously identified the transcription factor TBX3 as a tissue-specific component of the Wnt/β-catenin nuclear complex during mouse forelimb development. In this study, we show that TBX3 is functionally active in human colorectal cancer (CRC). Here, genome-wide binding and transcriptomics analyses reveal that TBX3 regulates cancer metastasis genes in cooperation with Wnt/β-catenin. Proteomics proximity labeling performed across Wnt pathway activation shows that TBX3 engages with several transcription factors and chromatin remodeling complexes found at Wnt responsive elements (WRE). Protein sequence and structure analysis of TBX3 revealed short motifs, including an exposed Asn-Pro-Phe (NPF), that mediate these interactions. Deletion of these motifs abrogates TBX3’s proximity to its protein partners and its ability to enhance the Wnt-dependent transcription. TBX3 emerges as a key modulator of the oncogenic activity of Wnt/β-catenin in CRC, and its mechanism of action exposes protein-interaction surfaces as putative druggable targets.

## Significance

Dysregulated Wnt signaling is a well-established driver of colorectal carcinogenesis. However, its pivotal role in normal intestinal stem cell homeostasis has posed significant challenges for its therapeutic inhibition, highlighting the need for discovering cancer-specific mechanisms. Our research reveals that the transcription factor TBX3, long recognized for its role in limb and heart embryogenesis, becomes aberrantly present in colorectal cancers (CRC). In this extraneous context, TBX3 adopts new functions: Instead of binding the DNA as a classic transcription factor, it employs short, conserved motifs on its surface to stick to other protein complexes—including the Wnt machinery—and fosters metastasis-inducing genetic programs. We hypothesize that targeting TBX3 could impair this aberrant embryonic-like behavior without affecting adult homeostasis.

As a highly conserved cell-to-cell communication mechanism, the Wnt/β-catenin signaling pathway coordinates a wide spectrum of processes by activating the expression of Wnt target genes (1). Wnt/β-catenin assumes a crucial role in nearly all facets of embryonic development and adult stem cell homeostasis, spanning pluripotency, proliferation, and differentiation (2). Its aberrant activation has been linked to many diseases such as developmental irregularities and various severe forms of cancer (3). A prime example of a disease largely driven by uncontrolled activation of the Wnt/β-catenin signaling pathway is colorectal cancer (CRC), ranking among the most lethal malignancies in humans (4). However, while much work has been dedicated to uncovering effective therapeutics to block oncogenic Wnt signaling, such interventions have been proven to be challenging because of the ubiquitous activity of Wnt signaling throughout the adult body and the difficulty in finding a suitable molecular target (5–8). Furthermore, aiming to intervene with β-catenin, the central regulator of this pathway, has presented challenges due to the rigorous control by a complex network of feedback mechanisms (1, 9, 10).

WNT ligands activate an intracellular cascade that culminates in the accumulation of β-catenin and its nuclear translocation in the nucleus (11). Nuclear β-catenin binds the TCF/LEF transcription factors positioned at Wnt responsive elements (WRE) on the chromatin together with transcriptional cofactors that include BCL9, its paralog BCL9L, PYGO1/2 (12–14), the ChiLS [Chip/LDB (LIM domain-binding protein) and a tetramer of SSDP (single-stranded DNA–binding protein, also known as SSBP) (15)] along with the members of the SWI/SNF (BAF) complex and the RNA polymerase II associated factors (16, 17).

However, a model in which a universal Wnt/β-catenin transcriptional complex drives the expression of Wnt target genes fails to explain a growing body of in vivo evidence. For instance, contrasting the notion of constitutive requirement for BCL9 and PYGO proteins for β-catenin nuclear activity, deletion of the genes encoding for these cofactors, in the mouse, causes later embryonic lethality and phenotypes that are significantly different from those caused by mutations in *Ctnnb1*, the gene encoding for β-catenin (18–21). Analogously, different cancer contexts characterized by high Wnt signaling display different requirements for these factors. For instance, both BCL9 and PYGO proteins play an important role in promoting epithelial-to-mesenchymal transition (EMT) in breast tumors (22–25). In contrast, BCL9 is involved in neoplastic lesions and EMT of the colorectal epithelium whereas PYGO only seems to play a minor or no role (26–29). Hence, the context-specific elements of the Wnt transcriptional apparatus are still unclear. We consider it imperative to delve into their discovery in order to precisely tailor our approach to modulating Wnt signaling in the context of CRC (30).

We have previously identified the developmental transcription factor TBX3 as a participant of the Wnt-mediated transcriptional regulation, serving as a PYGO-independent cofactor for the BCL9/9L adaptor proteins in developing mouse forelimbs (29). In this context, TBX3 displayed a bimodal behavior: while some TBX3 genomic binding sites were independent from Wnt/β-catenin and were accompanied by the classical TBX consensus sequence on the DNA, more than two-thirds of its genome-wide distribution displayed TCF/LEF binding consensus (29). This, and the fact that in these regions TBX3 binding to chromatin was abrogated when BCL9/9L and/or β-catenin were mutated (29), was evidence for TBX3 association to WREs as cofactor rather than a DNA-binding protein.

Here, we show that the role of TBX3 as Wnt signaling component goes beyond mouse embryogenesis. First, *TBX3* is expressed in cells derived from CRC patients, CRC cell lines, and other Wnt-driven cancers. Second, in this context TBX3 possesses a distinct genome-wide binding profile, which overlaps greatly with that of β-catenin, and controls the expression of genes that mediate metastatic transformation. Third, TBX3 associates to the Wnt/β-catenin transcriptional complex via protein–protein interactions that are mediated by evolutionarily conserved domains, including a Asn-Pro-Phe (NPF) tri-aminoacidic motif located at the C-terminus of the T-box binding domain. Deletion of the NPF and of the other motifs results in the loss of TBX3 protein interactions and abrogates the ability of TBX3 to enhance the Wnt/β-catenin-mediated transcription. This work therefore establishes TBX3 as a relevant player in CRC cell behavior and reveals interaction surfaces that could be targeted for future CRC molecular therapy.

## Results

### TBX3 Is Expressed in Human CRC Cells Together with Wnt Target Genes and Wnt Pathway Components.

**Fig. 1.**
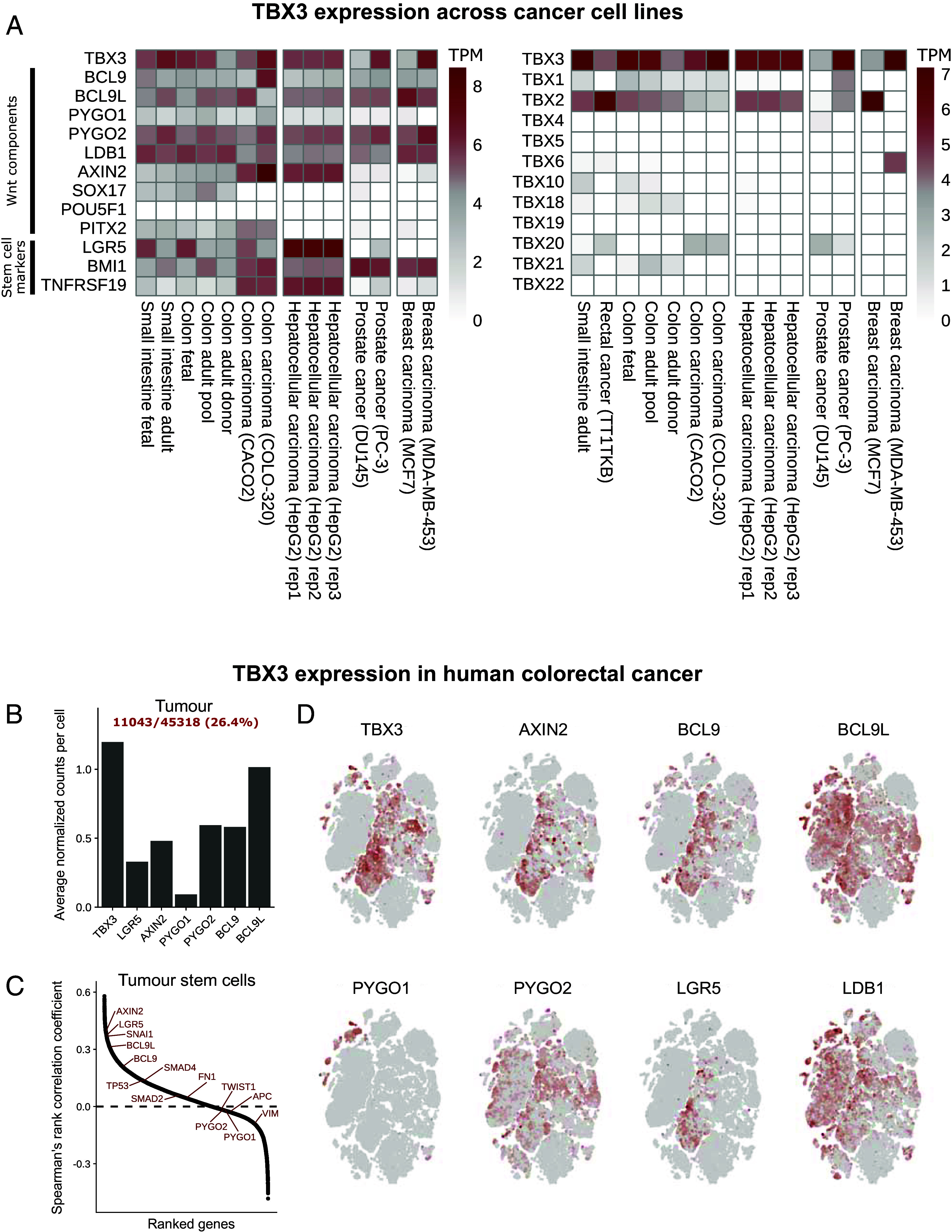
*TBX3* expression in human colorectal cancer (CRC). (*A*) Heatmaps showing normalized expression of *TBX3* for a set of cancer cell lines. *Left* panel: expression of *TBX3* and selected Wnt-relevant genes (including targets and components) of interest. *Right* panel: expression of all T-box transcription factors. CAGE-seq data generated by the FANTOM5 project and datasets were retrieved through the FANTOM5 Table Extraction Tool (https://fantom.gsc.riken.jp/5/tet/#!/search/hg19.cage_peak_ann.txt.gz). Expression values are given as tags per million (TPM) after adjustment for library size by the relative log expression method. (*B*) Barplot showing average normalized counts detected in tumor tissue cells for *TBX3* and the selected Wnt-relevant genes *LGR5*, *AXIN2*, *PYGO1*, *PYGO2*, *BCL9,* and *BCL9L*. (*C*) Ranked distribution of calculated Spearman’s rank correlation coefficients between *TBX3* and each other gene, based on cells identified as stem cells in tumor. The location of genes of interest are annotated with red colored text in the plot. (*D*) t-distributed stochastic neighbor embedding (t-SNE) plots of tumor cells highlighting the expression pattern across cells. scRNA-seq data from Uhlitz and colleagues (38).

We have previously identified TBX3 as a tissue-specific physical component of the Wnt/β-catenin transcriptional complex during mouse forelimb development (29). Moreover, while we showed that TBX3 overexpression in CRC cells could enhance their metastatic behavior, our former study did not investigate whether TBX3 was endogenously expressed in human CRC cells. Gene expression analysis of patient-derived human cancer cell lines obtained from the FANTOM 5 Consortium (31) showed that *TBX3* is indeed expressed in several tumor types of endo- and mesodermal origin, including, prominently, tumors from the gastrointestinal tract and the liver (Fig. 1 *A*, *Left* panel). It is likely that *TBX3* transcript levels are elevated in these types of tumors as a consequence of high Wnt/β-catenin signaling, as TBX3 is a bona fide Wnt target gene (our data and ref. 32). However, *TBX3* expression only partially overlapped with that of other Wnt target genes such as *AXIN2* and *LGR5* (Fig. 1 *A*, *Left* panel), suggesting varying levels of Wnt activation across tumor types, and mechanisms of uncoupled regulation between *TBX3* and other targets depending on the context (33). When considering the family of TBX transcription factors, TBX3 exhibited the highest expression level across the tumor cell types considered (Fig. 1 *A*, *Right* panel), supporting the dual nature of this protein as not only regulator of developmental processes, but also of tumorigenesis (34). We found that other notable alternative β-catenin cofactors, such as SOX17, expressed during endoderm formation (35), OCT4, present in mouse pluripotent stem cells (36), and PITX2 relevant in the ectoderm derived oral epithelium (37), were barely or not expressed across the models considered (Fig. 1), supporting the notion that TBX3, in this context, might play the more important role.

Single cell RNA sequencing (scRNAseq) of human CRC confirmed *TBX3* expression in tumor cell populations corresponding to a considerable 26.4% of all tumor cells (38) (Fig. 1*B*). Average normalized counts for *TBX3* correlated with counts of i) relevant Wnt targets (*AXIN2* and *LGR5*) that are also markers of intestinal stem cells, ii) genes encoding for Wnt/β-catenin signaling components (e.g., *PYGO1*, *PYGO2*, *BCL9,* and *BCL9L*) and mesenchymal markers associated to metastasis (e.g., *SMAD4*, *FN1*, *TWIST*) (Fig. 1*C*). Across tumor cell populations, *TBX3* expression significantly overlapped with that of *BCL9* and *BCL9L* in individual cells (Fig. 1*D*). Notable was also the expression of *TBX3* in the *LGR5^+^* cell compartment (Fig. 1*D*), indicating that TBX3 is likely to function in intestinal epithelial stem cells or cancer stem cells originating from this tissue (39).

### TBX3 Displays Genome-Wide Physical Association with the Chromatin in Human CRC Cells.

**Fig. 2.**
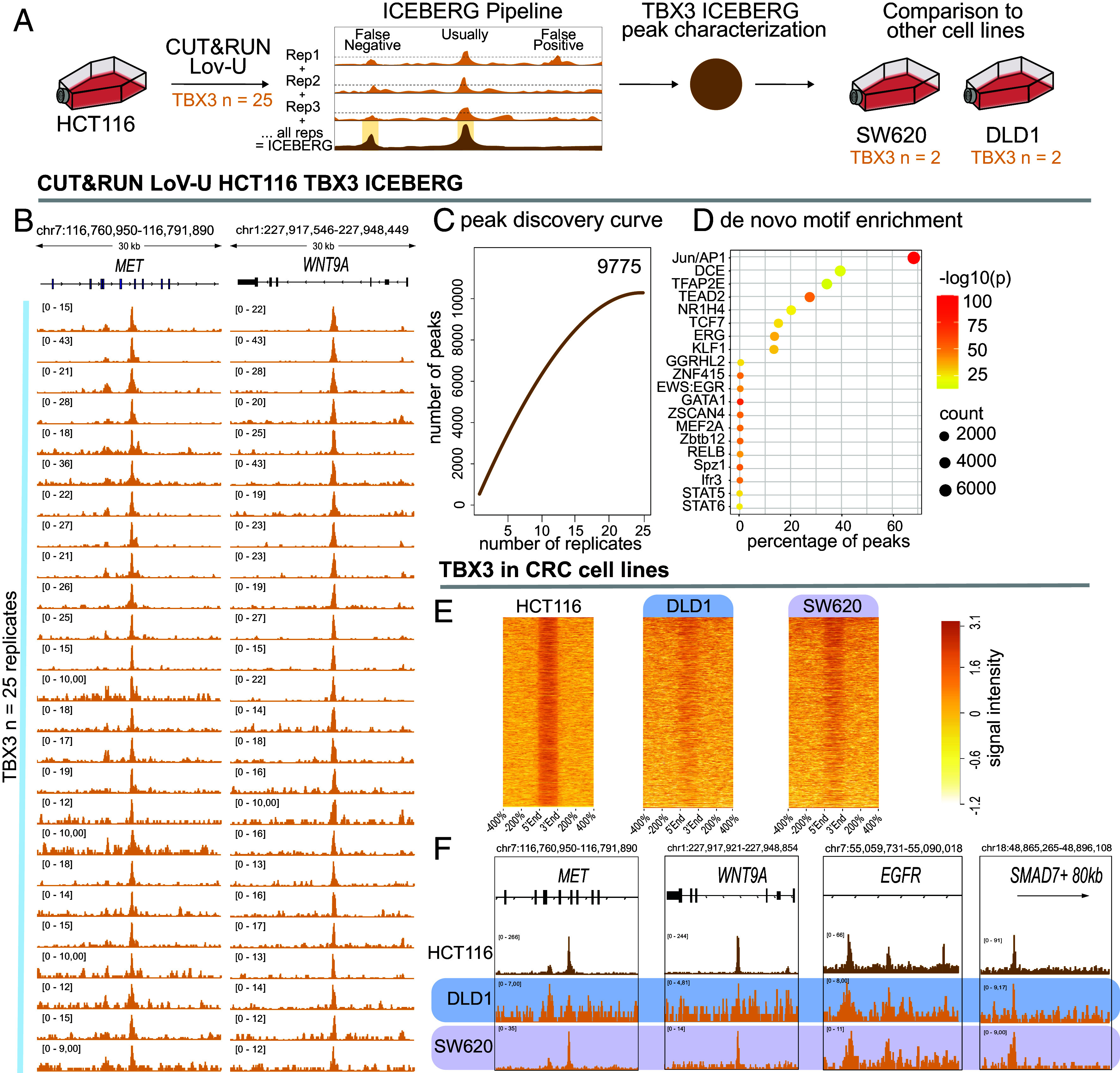
Genome-wide chromatin binding of TBX3 in CRC. (*A*) Schematic representation of experimental design. 25 replicates of CUT&RUN LoV-U were performed against TBX3 in HCT116 human CRC cells. The ICEBERG pipeline was used to process data, which was then compared to two other CRC cell lines: SW620 and DLD1. Briefly, reads are randomly sampled three times from all replicates in equal proportions and pooled to create aggregate datasets. These aggregates undergo peak calling and subsequent overlapping with the criteria of being present in at least two of the three aggregates and in at least one individual replicate (*P* < 0.01). (*B*) Genome browser tracks showing high signal to noise ratio for all 25 TBX3 replicates at the *MET* and *WNT9A* loci. (*C*) Polynomial regression curve of the rate of peak discovery in the TBX3 ICEBERG, which plateaus between 20 and 25 replicates. (*D*) De novo motifs/consensus sequences discovered by HOMER in the TBX3 peaks [q(Benjamini–Hochberg) < 0.0001 for all motifs displayed]. The TBX3 peaks are statistically devoid of enrichment for any TBX consensus. (*E*) Signal intensity plots of TBX3 CUT&RUN signal within ICEBERG peaks spanning the peak regions (from their 5’ to 3’ ends) and a ±400% across all peak regions, comparing HCT116 with DLD1 and SW620 cells. SW620 more closely recapitulates the signal of HCT116, though DLD1 also shows signal enrichment in most regions. (*F*) HCT116, DLD1, and SW620 TBX3 CUT&RUN genome browser tracks at the example loci *MET, WNT9A, EGFR,* and *SMAD7.* CRC = colorectal cancer.

We set out to determine the activity of TBX3 as regulator of gene expression by examining its genome-wide binding pattern in the human CRC cell line HCT116, in which a gain-of-function mutation in *CTNNB1*, the gene encoding for β-catenin, causes constitutive activation of Wnt signaling. We employed CUT&RUN (C&R) with Low-Volume and Urea [LoV-U; (40)] and performed 25 experimental replicates to define the full set of TBX3 binding sites in human CRC cells via the ICEBERG approach (41) (Fig. 2*A*). When individually inspected, all 25 C&R-LoV-U experiments appeared successful, as they invariably displayed high-signal peaks in previously identified TBX3 target genes (e.g., *WNT9A*, ref. 29). Importantly, all replicates pointed to the identification of previously unknown TBX3 bound regions, including the relevant proto-oncogene *MET*, whose mutations have been associated to aggressive forms of familial CRC (42, 43) (Fig. 2*B*). Our approach yielded a total of 9,775 high-confidence peaks, with peak discovery rate decreasing between replicates 20 and 25 (Fig. 2*C*), suggesting saturation of new finding and implying that our strategy is close to identify the complete set of TBX3 binding events in HCT116 cells (41). These sites were characterized by high signal-to-noise ratio and were mostly found in intergenic regions (~38%) and introns (~53%), while a smaller fraction (~5%) was located at promoter regions (*SI Appendix*, Fig. S1).

Motif/consensus analysis of the DNA sequences underlying the TBX3 binding sites revealed a plethora of transcription factor signatures, including those for the TCF/LEF, TEAD, and Jun/AP1 regulators (Fig. 2*D*). Surprisingly, we could not identify statistical enrichment for TBX consensus, suggesting that, in this context, TBX3 likely plays a role as a cofactor hijacking preassembled transcriptional complexes rather than directly binding to DNA. When C&R-LoV-U against TBX3 was performed in other human CRC cells, such as DLD1 and SW620, that instead of activating mutations in *CTNNB1* carry loss of function alleles of *APC* (44), comparable enrichment for TBX3 signal was observed (Fig. 2 *E* and *F*). As these CRC cell lines have been shown to be representative models of the main molecular subtypes or primary CRC, both concerning their mutational profile and their gene expression (45), our ICEBERG dataset of TBX3 target regions in different CRC cell types likely represents the general genomic activity of TBX3 in human CRC.

### The TBX3 Binding Profile Largely Overlaps with that of the Wnt Signaling Mediator β-Catenin.

**Fig. 3.**
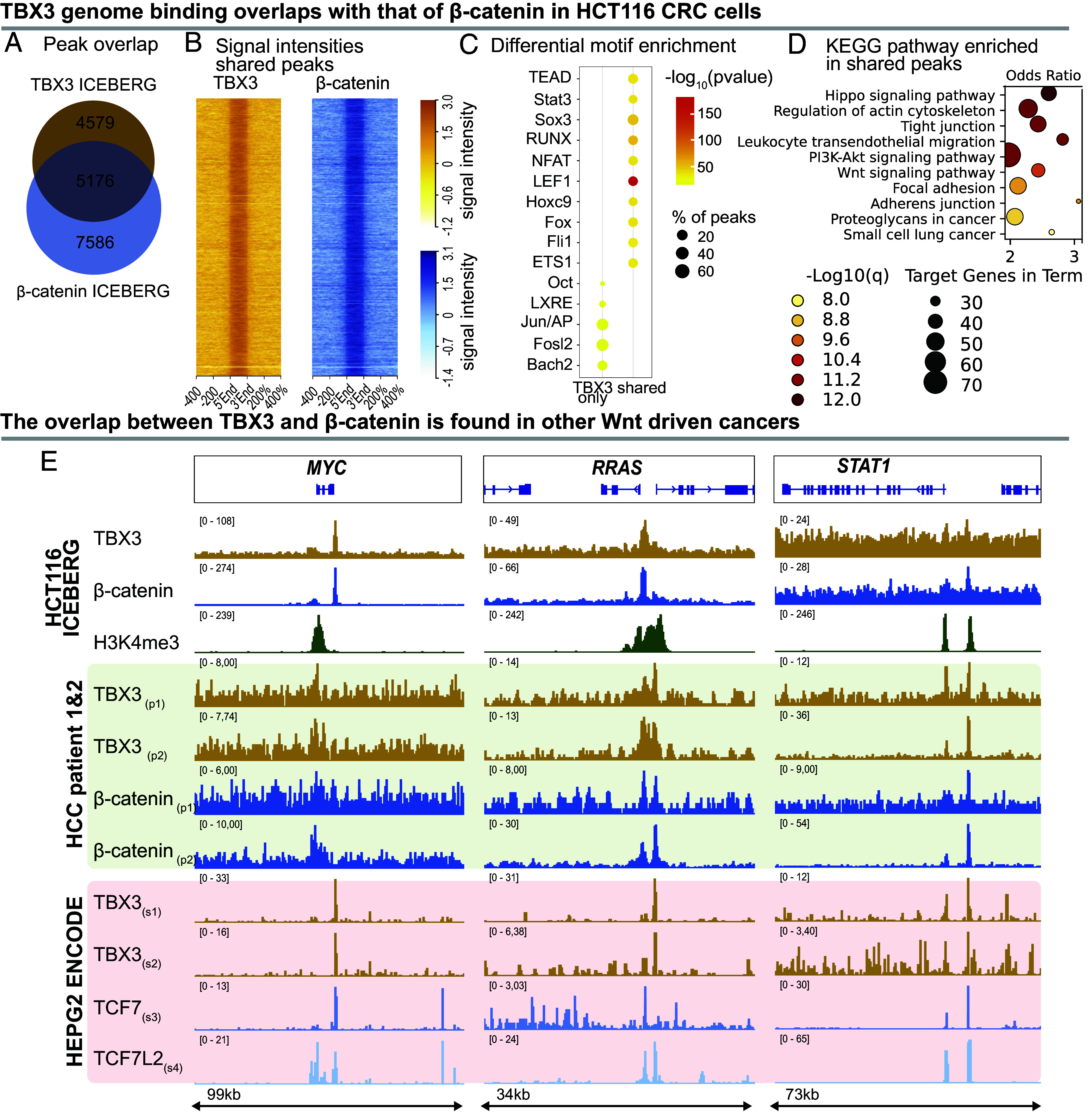
TBX3 and β-catenin have a partially overlapping binding profile in cancer. (*A*) Venn diagram depicting the overlap between ICEBERG peak regions of TBX3 and β-catenin in HCT116 cells. Over 50% of TBX3 peaks are also bound by β-catenin. (*B*) Signal profiles of TBX3 and β-catenin CUT&RUN signal at shared peaks, ranked based on the TBX3 condition. (*C*) Differential motif/consensus enrichment done with HOMER between TBX3 only peaks and shared peaks. q(Benjamini–Hochberg) < 0.0001 for all motifs displayed. TBX3 only peaks are differentially enriched for Jun/AP1 family factors, while shared peaks are highly significantly enriched for the Wnt signaling transcription factor LEF1, as well as others. Neither set individually shows statistical enrichment for TBX motif/consensus. Color indicates enrichment *P*-values, dot-size indicates the percentage of peaks enriched for respective motif. (*D*) Top ten KEGG pathways enriched (odds ratio, background all protein coding genes) in TBX3 and β-catenin shared peak associated gene sets. Shared peaks are enriched for the Wnt and Hippo pathways, for general pathways in cancer, and for focal adhesion. Dot-size indicates the gene count, color indicates the negative decadic logarithm of the q-value after multiple testing correction (Benjamini–Hochberg False Discovery Rate adjustment). (*E*) *Upper* panel: The *MYC*, *RRAS*, and *STAT1* loci serve as examples of overlapping signals in TBX3 and β-catenin CUT&RUN ICEBERGs in HCT116 cells (orange and dark blue tracks, respectively). Green tracks represent H3K4me3 enrichment, marking active promoter regions of the highlighted genes. *Middle* panel (highlighted in green): TBX3 (orange) and β-catenin (dark blue) CUT&RUN tracks at the same loci, derived from two distinct patient-derived primary hepatocellular carcinoma (HCC) biopsies (p1/2 = patient 1 or 2). *Lower* panel (highlighted in red): Publicly available ChIP-seq data from ENCODE, derived from HEPG2 cells, displaying TBX3 (orange; s1 = ENCFF287XCG, s2 = ENCFF397PQZ), TCF7 (blue, s3 = ENCFF371KMF), and TCF7L2 (light blue, s4 = ENCFF406WNX) tracks at the same loci.

From these and previous results obtained in the embryonic murine forelimb (29), we hypothesized that TBX3 could cooperate with β-catenin also in CRC cells. This hypothesis would imply that the TBX3 bound regions in CRC cells (Fig. 2) would match, to a certain extent, β-catenin occupancy. To test this, we measured the peak overlap identified by the TBX3 ICEBERG with those found by the β-catenin ICEBERG [also 25 replicates in HCT116, (41)]. Indeed, 53% of the identified TBX3 binding sites (5,176 out of 9,775 peaks) were also bound by β-catenin, indicating that more than half of TBX3 genome-wide activity is in concert with the Wnt/β-catenin transcriptional complex (Fig. 3*A*). Even the TBX3-only and the β-catenin-only peaks displayed minor signal enrichment for the other factor—β-catenin or TBX3, respectively (*SI Appendix*, Fig. S1*C*), suggesting that our stringent peak calling parameters and overlap definition (at least one nucleotide in common) might lead us to underestimate an even stronger interplay between TBX3 and β-catenin. The high-confidence TBX3-β-catenin common peaks (Fig. 3*B*) exhibit the strongest signal when compared to those that are, according to peak calling, exclusive to one or the other factor (*SI Appendix*, Fig. S1*D*).

Genomic annotation of the TBX3-bound regions by HOMER identified prominent association with intergenic and intronic regions and found no obvious differences between the locations where TBX3 binds alone versus those shared with β-catenin (*SI Appendix*, Fig. S1*A*). TBX3-only peaks are enriched for Jun/AP1 family factors, among others, while shared TBX3 and β-catenin peaks presented enrichment for the Wnt signaling transcription factor LEF1 (Fig. 3*C*). The absence of TBX binding consensus appearing in this analysis, in conjunction with the enrichment of the TCF/LEF signature, suggested that also in CRC cells, as previously found in developing mouse forelimbs (29), the association of TBX3 to these loci depends on the physical interaction of TBX3 with components of the Wnt/β-catenin transcriptional complex. KEGG pathway analysis of genes associated to peaks that present both TBX3 and β-catenin binding showed enrichment for biological processes related to regulation of cell migration and proliferation, including remodeling of actin cytoskeleton, focal adhesions, and the Hippo, Ras, and Wnt signaling pathways (Fig. 3*D*) [pathways were considered significant with a q-value < 0.05 after multiple testing correction (Benjamini–Hochberg FDR adjustment), displayed are the top 10 hits], raising the possibility that TBX3 is involved in the direct regulation of these groups of genes in CRC.

Of note, overlapping genomic occupancy between TBX3 and β-catenin at relevant cancer-inducing genes, such as *MYC*, *RAS,* and *STAT1* is also found in hepatocellular carcinoma (HCC) cells, both in cell lines (HepG2 from ENCODE; see Methods) and in two independent patient-derived HCC biopsies (our data) (Fig. 3*E*). This suggests that the cooperation of TBX3 with Wnt signaling may be a more general feature of Wnt-driven cancers.

### TBX3 Regulates Genes that Promote CRC Cell Metastatic Behavior.

**Fig. 4.**
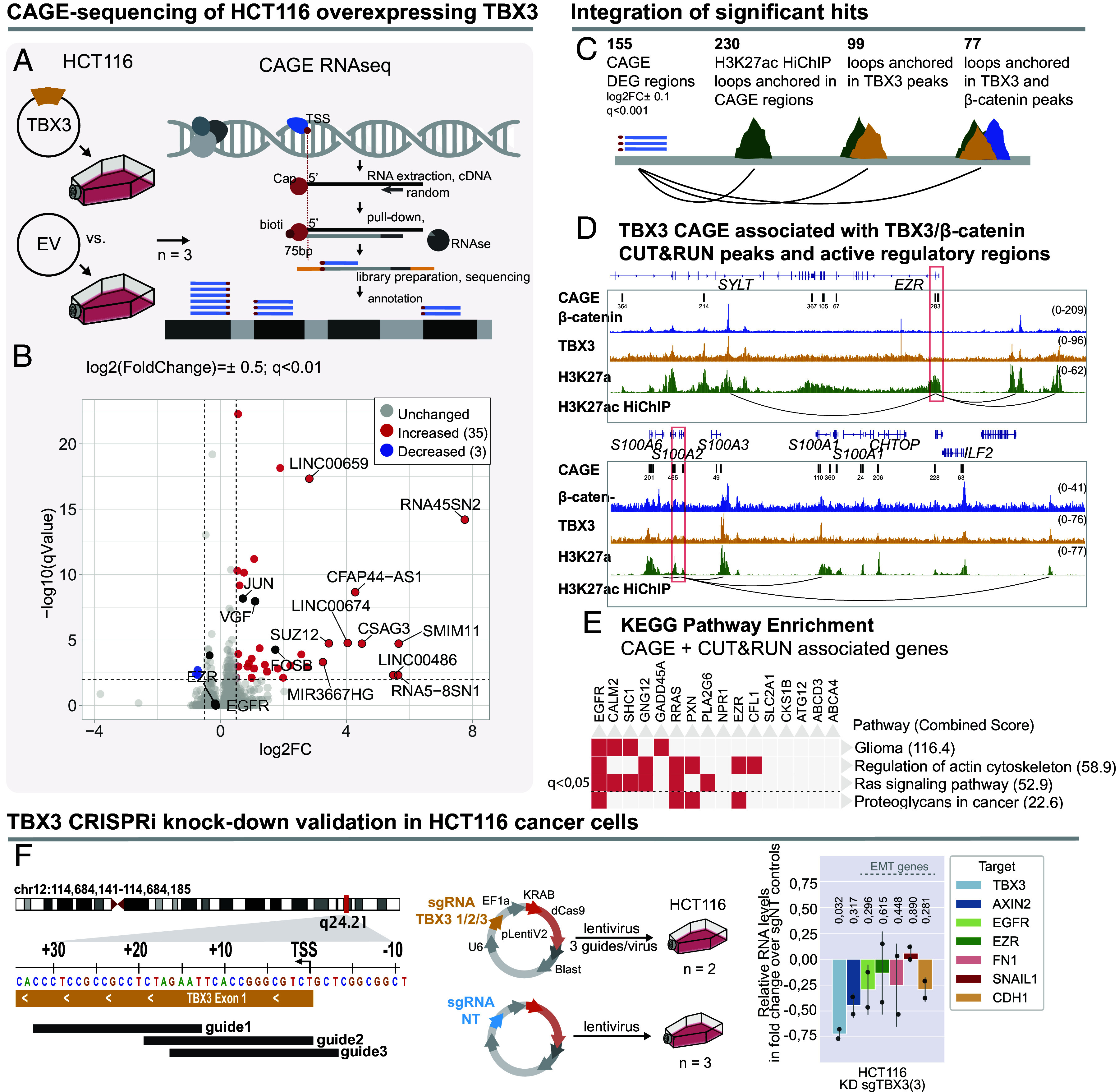
CAGE-seq identifies TBX3-regulated drivers of metastasis. (*A*) Schematic representation of the experimental setup, where *TBX3* and an EV-ctrl were overexpressed in HCT116 cells (n = 3 each), and the performed procedure for Cap-Analysis Gene Expression Sequencing (CAGE-Seq). CAGE-seq was used to identify both differentially expressed genes (DEG), their exact transcription start sites (TSS), and regions of transcribed enhancer RNAs. (*B*) Volcano plot of CAGE-seq DEG regions. Upregulated transcripts in blue (q < 0.01, log2FC > 0.5), down-regulated in red (q < 0.01, log2FC<-0.5), excluding *TBX3*. 38 genes were differentially expressed according to these criteria. (*C*) Schematic of the strategy used to identify genomic elements regulated by TBX3 and TBX3-β-catenin. H3K27ac-HiChIP data identified DNA–DNA connections between active regulatory regions, including promoters and putative enhancers. We focused on the differentially expressed RNAs (CAGE-seq, less stringent filtering criteria with *P* < 0.001 and q ± 0.01) that emerge from regions of the genome that are connected via H3K27ac-marked regions (green) to TBX3 (orange) and/or β-catenin (blue) HCT116 CUT&RUN peaks. (*D*) Example genome browser visualization of CAGE-seq DEG regions that emerge from regions that are connected via H3K27ac anchored chromatin loops to TBX3 and β-catenin shared CUT&RUN peaks, in cancer relevant loci near *S100* genes and *EZR.* (*E*) Top enriched KEGG pathways (rows) for genes associated with CAGE-seq DEG loops (input genes in columns), showing enrichment for glioma, actin cytoskeleton regulation, and the Ras pathway. The combined significance score across the human genome [log(p) * z-score (deviation from expected rank), *P*-value from Fisher’s exact test] is shown in brackets next to each term. The dotted line represents the q < 0.05 significance threshold, adjusted for multiple testing using the Benjamini–Hochberg method. (*F*) *Left*: Schematic illustration of the experimental approach of knocking down (KD) TBX3 with CRISPR inhibition (CRISPRi). Three guides targeting the proximity of TBX3s’ TSS were designed and cloned into a CRISPRi-KRAB lentiviral vector. Nontargeting (NT) guides were used as control. Three replicates for HCT116 were infected with virus containing the pooled sgTBX3 guides (3), or sgNT controls. *Right*: Relative expressions of *TBX3, AXIN2,* and a panel of EMT genes including *EGFR*, *EZR*, *FN1*, *SNAIL1*, *CDH1* were analyzed with reverse-transcription PCR, normalized to *GAPDH* and visualized as fold change over the average of the three NT-controls. One replicate was excluded due to insufficient reduction of TBX3 expression. An independent Student’s *t* test was used to compare KD with the NT control, respective *P*-values are indicated above each condition. *TBX3* showed significant downregulation (*P* < 0,05, n = 2), and other targets display (e.g., *AXIN2*, *EGFR*) a similar trend. Despite low statistical significance, likely given by n = 2, these are interpreted as a decrease in expression.

The genome-wide binding profile of TBX3 indicated that this protein might directly regulate genes involved in metastatic behavior (Figs. 2 and 3). This is important, as it could explain our previous observation that TBX3 overexpression in HCT116, which carries a constitutively active β-catenin, was sufficient to enhance metastatic cell dissemination in in vivo models (29). Moreover, others have identified that overexpression of TBX3 in CRC correlated with poor prognosis of CRC (46). To find the relationship between TBX3 physical chromatin-binding and the observed enhancement of metastatic cell behavior, we set out to measure which genes are transcriptionally controlled by TBX3 in CRC. We employed Cap-Analysis Gene Expression sequencing (CAGE-seq), which measures the abundance of RNAs and maps the transcriptional start sites (TSS) and other 5’-capped regulatory regions, that include putative regulatory elements (31, 47) (Fig. 4*A*). CAGE-seq revealed a total of 155 differentially expressed transcripts in HCT116 upon TBX3 overexpression compared to control empty vector (EV) condition (Fig. 4*B*): Of these, 129 were upregulated and 26 down-regulated, possibly indicating that, in this context, TBX3 primarily mediates gene activation rather than gene repression (48).

We set out to understand which of these changes in RNA abundance could be directly controlled by TBX3. To this aim, we integrated the TBX3 genomic distribution with the network of active CRC-enhancers obtained by HiChIP targeting the marker of open chromatin H3K27ac in HCT116 (49). The 155 differentially expressed CAGE-seq regions were anchored to 230 H3K27ac-mediated DNA–DNA interaction loops (Fig. 4*C*; note that each CAGE-seq region could be anchored to >1 H3K27ac loop), supporting the notion that they correspond to active regulatory elements (50). Among the many H3K27ac-ornamented regions looping into differentially expressed CAGE-seq RNAs, a large fraction (99 out of 230, 43%) was occupied by TBX3, and the majority of these (77 out of 99, 78%) was co-occupied by both TBX3 and β-catenin (Fig. 4*C*), indicating that TBX3 and β-catenin together promote the differential RNA production measured by CAGE-seq.

KEGG pathway enrichment analysis of the TBX3/β-catenin regulated genes showed high-fold enrichment for cancer-related processes (Fig. 4*D*). Integration of CAGE-seq, TBX3 C&R-LoV-U, β-catenin C&R-LoV-U, and HiChIP targeting H3K27ac permitted us to identify the specific instances of direct gene regulation by TBX3 and β-catenin in CRC. These include cancer relevant loci near the *S100* family of genes, which are considered prognostic markers for patients with colorectal neoplasia (51), and *EZR*, a known mediator of invasion and metastasis (52, 53) (Fig. 4*E*).

To validate the identification of direct targets, we devised a strategy to knock-down (KD) *TBX3* expression. We designed three sgRNAs spanning the *TBX3* promoter region (from −5 to ca. +30 in respect of the TSS; Fig. 4 *F*, *Left*) and coexpressed them with a dCas9-KRAB inhibitor complex in HCT116. This caused a 75% reduction in *TBX3* mRNA, with consequent downregulation of *AXIN2* (confirming the positive regulation of Wnt targets by TBX3) and of other relevant CRC metastasis genes (Fig. 4 *F*, *Right* panel). When comparing regulation of these direct targets with publicly available RNA-sequencing data of *TCF7L2* knock-out (KO) in HCT116 (54), we observe a similar trend in the expression of these TBX3 targets (*AXIN2* log2FC = −0.094, padj. = 0.826; *EGFR* log2FC = −0.519, padj. = 0.289; *EZR* log2FC = −0.14, padj. = 0.35; *FN1* log2FC = −0.576, padj. < 0.001). While this supports the notion that TBX3 may regulate genes in collaboration with the Wnt transcriptional machinery, we cannot exclude that other TCF/LEF factors could potentially counteract this effect.

### TBX3 Physically Associates with the Wnt/β-Catenin Transcriptional Complex.

**Fig. 5.**
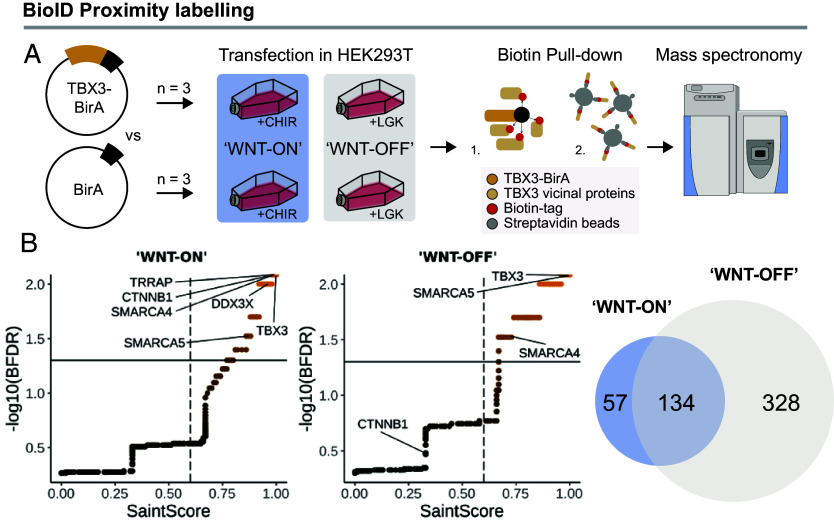
BioID reveals the effect of Wnt activation on TBX3 protein-proximity network. (*A*) Schematic representations of the experimental design. HEK293 cells expressing a TBX3-fused biotin ligase (BirA) versus an empty vector-BirA, were cultured in either 10 µM CHIR99021 (“WNT-ON”) or 10 nM LGK974 (“WNT-OFF”) supplemented media (n = 3). Biotinylated proteins were pulled down and further subjected to mass spectrometry analysis. (*B*) *Left* panel: scatter plots displaying the mass spectrometry results of TBX3-BirA expressing cells in “WNT-ON” and “WNT-OFF” conditions over the EV-BirA control to distinguish from background contaminations, expressed as SaintScore (thresholds refer to SaintScore > 0.6, BFDR < 0.05). *Right* panel: Venn diagram indicating common (134) and unique (328 for “WNT-OFF,” 57 for “WNT-ON”) labeled proteins in both conditions.

In our motif analysis we noted the absence of TBX consensus sequences. We reasoned that TBX3 in CRC might act via hijacking other chromatin-associated protein complexes. Perhaps, these DNA-binding sites that characterize TBX3’s genome association during development, might be inaccessible in other, nondevelopmental cellular contexts. Hence, we set out to screen for TBX3-interacting nuclear proteins. We selected the Wnt-responsive HEK293T cells, which allowed us to modulate Wnt/β-catenin signaling either by the GSK3 inhibitor CHIR99021 (CHIR) to activate the pathway (WNT-ON) (55) or by the Porcupine inhibitor LGK974 (LGK) to inhibit the endogenous secretion of WNT ligands (WNT-OFF) (56), and employed the BioID technology (57) to map the physical proximities of TBX3 in both conditions (Fig. 5*A* and *SI Appendix*, Fig. S2). TBX3 was cloned in-frame to the biotin-ligase BirA and expressed in HEK293T cells cultured in WNT-ON and WNT-OFF. Biotinylated nuclear extracts were subjected to streptavidin pulldown, and nuclear proteins in the vicinity of TBX3 were isolated and identified via mass spectrometry. A total of 191 candidate interactors were found in CHIR-treated cells and 462 candidate interactors were found in LGK-treated cells (SAINT score > 0.6, BFDR < 0.05) (Fig. 5*A*). Among the proteins that were significantly enriched in CHIR-treated cells we identified several known regulators of gene expression associated to Wnt signaling, such as SMARCA4 and SMARCA5, part of the SWI/SNF (BAF) chromatin remodeling complex (16), DDX3X (58), TRRAP (59), as well as β-catenin (CTNNB1 in Fig. 5*B*). Activation of Wnt signaling by CHIR seemed to enhance the vicinity of TBX3 to all these proteins (Fig. 5 *B*, *Left* panel), possibly reflecting the broad stabilization of the proteome that follows GSK3 inhibition (60). In WNT-OFF conditions, many of these interactions are reduced but still observed, except for that with β-catenin (Fig. 5 *B*, *Middle* panel). These experiments support a model in which TBX3 is tethered to the WREs by direct and promiscuous physical contacts with members of the Wnt/β-catenin transcriptional complex during Wnt pathway activation.

### Conserved Three-Amino Acid Motifs Mediate TBX3 Interactions to the Wnt/β-Catenin Transcriptional Complex.

**Fig. 6.**
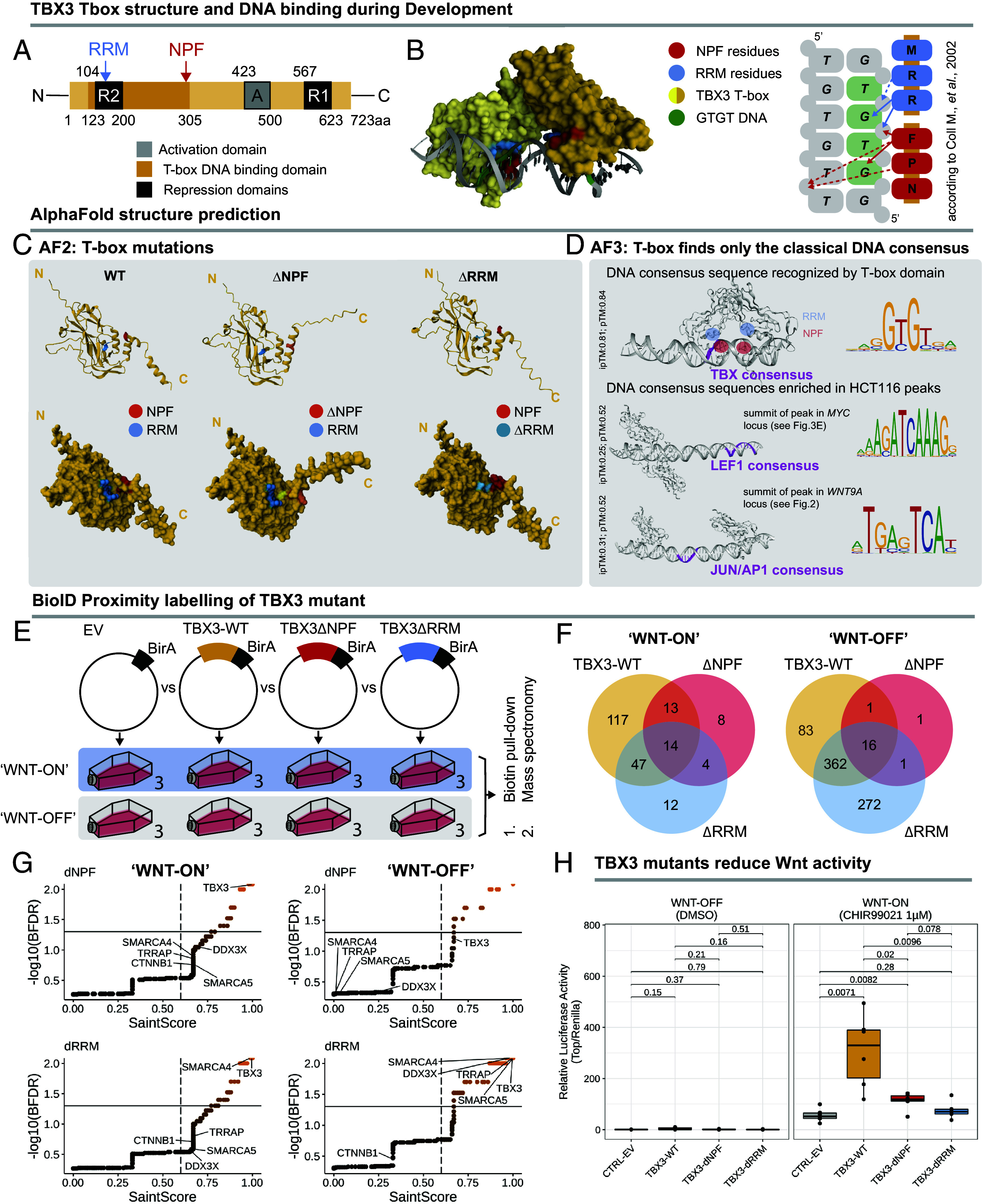
TBX3’s DNA-binding domain engages in protein–protein interactions. (*A*) Illustration of the relative location of TBX3’s T-box DNA-binding domain in respect to the three-amino acid motifs NPF and RRM. (*B*) 3D structure of the previously published, experimentally determined, DNA-bound T-box domain (PDB 1H6F) dimer of human TBX3 (63) with the NPF motif highlighted in red and the RRM motif highlighted in blue. The illustration to the left shows how both motifs (and only those) convey sequence specificity for recognizing the GTGT core consensus DNA sequence typical of TBX factors. Normal arrows indicate polar interactions, dashed arrows indicate hydrophobic interactions. (*C*) AlphaFold2 predictions of the TBX3’s T-box (plus 20 additional amino acid residues) with and without deletion of NPF or RRM. The corresponding areas are in red and blue, respectively. (*D*) AlphaFold3 predictions of protein–DNA interactions. Models were obtained using two T-box protein sequences and dsDNA containing DNA consensus sequences enriched in developmental [DNA fragment from Coll et al. (63) or cancer contexts. For the latter, peak summit regions of the TBX3 C&R ICEBERG in HCT116 falling into the *MYC* (see Fig. 3*G*) or *WNT9A* (see Fig. 2*B*) regulatory locus were scanned for the LEF1 or Jun/AP1 consensus motif using FIMO. (*E*) Schematic illustration of the experiment investigating changes in TBX3 vicinal proteins upon deletion of its NPF(TBX3-∆NPF) or RRM (TBX3-∆RRM). HEK293T cells were transfected with either EV-BirA as a control or TBX3-WT-BirA, TBX3-∆NPF-BirA, TBX3-∆RRM-BirA, in “WNT-ON” (10 µM CHIR99021) and “WNT-OFF” (10 nM LGK974) conditions (n = 3). Biotinylated proteins from the pull-down were further subjected to mass spectrometry analysis and visualized subtracting hits overlapping with the EV-BirA control. (*F*) The number of common and unique vicinal proteins of TBX3 wild-type and both mutants in “WNT-ON” (*Left*) and “WNT-OFF” (*Right*) conditions, visualized as Venn diagrams (SaintScore > 0.6, BFDR < 0.05). (*G*) Top ranking protein proximity hits of the TBX3 mutants in both conditions over the respective EV-BirA control to distinguish from background contaminations, expressed as SaintScore (thresholds refer to SaintScore > 0.6, BFDR < 0.05). (*H*) TOPFlash assay investigating the effect of both deletions on the activation of Wnt/β-catenin signaling. HEK293T cultured in 10 nM LGK, were transfected with a control empty vector (EV), TBX3-WT-BirA, TBX3-BirA-∆NPF, or TBX3-∆RRM-BirA. All cells were transfected with the TCF/LEF-luciferase reporter SuperTOPFlash and Renilla control plasmids. WNT-OFF (*Left* panel) and WNT-ON (*Right* panel) conditions were tested with 6 replicates per condition. Activation of Wnt signaling was measured as relative luciferase activity. Data are visualized as box and whiskers plots. A Student’s *t* test was used for pairwise comparisons of groups, and significance values (*P*-value) are displayed above the boxes. A *P*-value < 0.05 was used to determine statistical significance.

Investigation of the amino acid residues composing TBX3 revealed an NPF (Asparagine-Proline-Phenylalanine) motif at the edge of the DNA-binding T-box domain (Fig. 6*A*). The NPF caught our attention for three reasons. First, the NPF motif was among the most evolutionarily conserved parts of TBX3 and across other members of the TBX family of transcription factors (*SI Appendix*, Fig. S3*A*). Second, an NPF motif was first described as the transactivation domain of the Wnt cofactor Pygopus (PYGO1 and PYGO2 in humans) and recent structural work revealed that the PYGO-NPF mediates its interaction with the Wnt/β-catenin transcriptional complex (61, 62). Third, our original identification of TBX3 as a facultative member of the Wnt/β-catenin transcriptional complex derived from genetic data implying TBX3 function in PYGO-independent phenotypes (29).

In our analysis, we also considered another three-amino acid motif, the RRM (arginine–arginine–methionine), that was previously identified as constitutive mediator of the DNA-binding domain of TBX3 (63). Structurally, the NPF and the RRM are situated opposite each other, with a pocket between them that accommodates the minor groove of the DNA helix (Fig. 6*B*). Both NPF and RRM can form direct polar bonds with the nucleotides of the DNA TBX GTGT core consensus, and hydrophobic bonds with the DNA backbone, suggesting similar DNA-binding contributions [Fig. 6*C*, (63)]. Compared to the RRM, the NPF is an exposed “anchor” between two alpha-helices at the C-terminus of the T-box domain, and could in principle constitute a docking site for other proteins. AlphaFold2 (AF2) predicts that their mutations cause local, and not global, 3D structural perturbations: Deletion of the NPF leads to a rotation of the C-terminal domain (Fig. 6 *C*, *Middle* panel), while deletion of the more inward-located RRM narrows the gap between the two regions (Fig. 6*C* and *SI Appendix*, Fig. S3*B*). The reliability of AF predictions (64), together with the comparable amounts of full-length versus mutant TBX3 variants detected, increase our confidence that these small deletions do not perturb general protein folding.

As the NPF and RRM are part of the DNA-binding domain of TBX3 (Fig. 6*B*), it remains to be determined whether, in CRC cells, these motifs drive the TBX3-DNA or the TBX3–protein interactions.

The genome-wide binding profile of TBX3 in HCT116 supports the latter explanation as the sequences underlying its binding positions do not contain the classic TBX consensus sequence, but rather that of other factors (e.g., TCF/LEF and Jun/AP1). In support of this view, while AlphaFold3 (AF3) correctly identifies the exact binding position of TBX3 on the GTGT core consensus, it fails to predict any interaction between TBX3 and DNA sequences that we found underlying TBX3 peaks in HCT116, containing LEF1 or Jun/AP1 consensuses (Fig. 6*D* and *SI Appendix*, Fig. S3*C*).

To test this experimentally, we cloned mutant versions of TBX3 lacking either the NPF or the RRM amino acid residues (TBX3∆NPF or TBX3∆RRM, respectively) in-frame with the BirA biotinylating enzyme and carried out a BioID assay similar to the one described above. TBX3∆NPF and TBX3∆RRM were expressed at comparable levels as TBX3-WT and were both detectable as top hits by mass-spectrometry upon transfection (Fig. 6*G*). In WNT-ON and WNT-OFF conditions, both mutations led to a dramatic reduction of the significant protein partners identification (Fig. 6*F*). Of note, both TBX3∆NPF and TBX3∆RRM were no longer vicinal to β-catenin in WNT-ON, supporting the notion that these mutations cause detachment of TBX3 from the transcriptional complex (Fig. 6*G*). Similarly, both mutations caused the loss of interaction with DDX3X, TRRAP, and with components of the chromatin remodeler SWI/SNF complex with the notable exception of SMARCA4, indicating selective contributions of the two TBX3 three-amino acid motifs. Whether the NPF connects TBX3 to the Wnt transcriptional complex via direct binding to these proteins, or if other factors may influence their proximity in vivo remains to be determined.

To test the consequence of impairing these interactions on transcription, we employed the transcriptional SuperTOPFlash reporter that specifically and exclusively allows DNA-binding of the TCF/LEF transcription factors (65). The absence of TBX binding sites on the SuperTOPFlash implies that TBX3 cannot act through its DNA-binding ability. TBX3-WT-BirA significantly boosted the transcription from the Wnt reporter SuperTOPFlash only upon Wnt pathway stimulation (Fig. 6*H*) (Student’s *t* test, *P* < 0.05). TBX3∆NPF-BirA and TBX3∆RRM-BirA, on the other hand, had a considerably reduced effect on SuperTOPFlash transcription (Fig. 6*H*) (Student’s *t* test, *P* < 0.05).

These experiments conclusively show that, in the context of WREs binding, the NPF and RRM motifs are responsible to mediate protein–protein interactions between TBX3 and the components of the Wnt/β-catenin transcriptional complex and not the interaction with the GTGT core consensus DNA sequence.

## Discussion

Revealing the molecular composition of transcriptional complexes is key to understand cell behavior during development, homeostasis, and disease. Our primary objective is to understand how the Wnt signaling pathway, with its apparent universal transduction mechanism, can drive diverse gene expression patterns across a wide range of developmental and homeostatic contexts (2, 66). This, we posit, will be key to unraveling what are the relevant cell-specific transducers that confer oncogenic potential to the Wnt/β-catenin axis, and broadly targeting the Wnt pathway can lead to undesirable consequences (67).

**Fig. 7.**
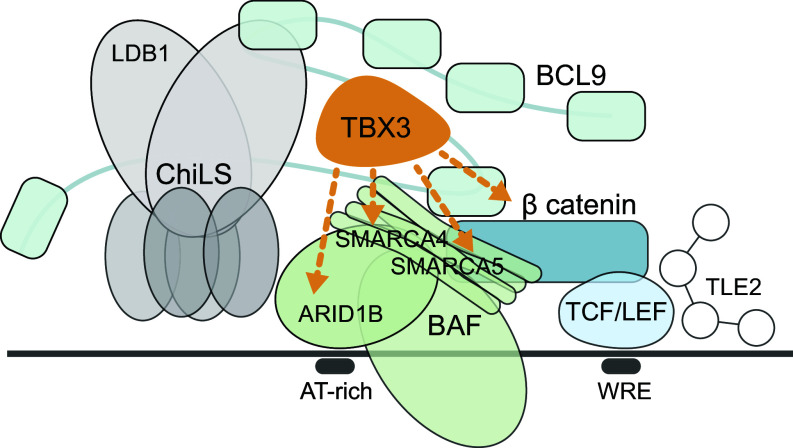
A schematic representation of TBX3’s interaction with the Wnt transcriptional complex. TBX3 interacts with β-catenin, core component of nuclear Wnt signaling (blue), and SMARCA4/ SMARCA5, components of the BAF chromatin remodeling complex (green). WRE = Wnt responsive elements.

We have previously discovered that the tissue-specific and human disease-relevant transcription factor TBX3 can assist the BCL9/β-catenin driven transcriptional complex downstream of Wnt signals during mouse forelimb development (29). Our finding opened the possibility that the physical interplay between TBX3 and the Wnt pathway (Fig. 7) might underlie the oncogenic activity that had been associated to its aberrant expression in CRC and other cancers (32, 34, 68, 69). In this study, we fulfill this prediction.

We find that TBX3 is expressed in subsets of CRC and HCC cells, likely as a consequence of hyperactive Wnt signaling, as its expression overlaps with that of other Wnt target genes and Wnt signaling components, as well as markers of the intestinal stem cells. Other previously identified β-catenin cofactors, including the endodermal transcription factor *SOX17*, appear not to be significantly expressed in these contexts, suggesting that in CRC and HCC it is more likely TBX3 that takes part of the Wnt transcriptional complex. Consistently, we detected TBX3 engaging with transcriptional complexes positioned genome-wide at regulatory sites along the chromatin, modulating the expression of genes that are known to drive CRC metastatic dissemination. The identification of the molecular activities of TBX3 in human CRC cells not only explains how its overexpression can increase their metastatic potential, as we previously observed, but also establishes TBX3 as a regulator of metastasis and as a putative target for molecular therapy. That TBX3, in CRC cells, interacts with the chromatin mostly via protein–protein interactions raises the possibility that TBX3’s function in other biologically relevant contexts, such as in pluripotent stem cells (70), is also partly mediated by its interplay with the protein complexes identified in the current study. It is possible that the developmental binding sites are inaccessible to TBX3 in other, nondevelopmental cellular contexts, such as CRC and HCC. Puzzling to us remained how the same domain of TBX3 could be responsible for interacting with both DNA and proteins. While additional studies are needed to clarify this from both a mechanistic and functional point of view, it is important to notice that other groups found that the DNA-binding T-Box is responsible for the interaction with proteins. The TBX factors Brachyury, Eomes, TBX3, TBX6, and TBX20 have been shown to interact with the homeodomain of MIXL1 precisely via their T-Box (71), and the T-box of TBX2, TBX3, and TBX5 was shown to mediate binding to MSX1 and MSX2 in cardiac cells (72). We consider these studies as an independent validation that lends credibility to our mechanistic explanation.

While more than 30 years of evidence have supported a critical role of abnormal Wnt/β-catenin activation as causative of CRC (73, 74), the field is still missing Wnt pathway inhibitors for therapeutic treatment of this type of cancer (5–8, 75). Identification of the TBX3 prominent activity in CRC generates the impetus for considering TBX3 as a potential molecular target. It remains to be established whether TBX3 is essential for intestinal stem cells homeostasis as β-catenin (76), or if its presence is dispensable as is the case for BCL9 and BCL9L (77). While we consider the latter hypothesis more plausible, since the phenotypes caused by loss of *Bcl9/9l* or *Tbx3* are similar in the mouse—an observation that drew us onto studying TBX3 in the first place—only functional or knockout in vivo experiments will provide a final answer. The identification of the NPF motif provides additional impetus to continue this investigation, also given its exposed position on the 3D structure of TBX3 (Fig. 6 *B* and *C*), and the effectiveness of its deletion in abrogating protein interactions—a significantly stronger effect than that of mutating other motifs such as the RRM (Fig. 6 *F* and *G*). Given its accessibility on the protein surface of TBX3 and its functional significance, the NPF could certainly constitute an attractive target for antibodies or small molecules. The NPF motif appears to be present in several other proteins and has a defined mechanism of action (78). Many of these proteins, such as Pygopus and ARIDB1, possess oncogenic activity (61), suggesting that generating protein-specific or general NPF inhibitors might be a worthwhile initiative.

Intriguingly, inspection of published data on protein–protein interactions in colon cancer cell lines revealed that significant interactions between TBX3 and β-catenin had already been detected (79); we consider this an important independent validation of our study. The same authors, in a previous prominent article, had identified an interplay between β-catenin and another member of the TBX family of transcription factors, TBX5, which together with YAP1 and β-catenin was essential to mediate survival and tumorigenesis of several β-catenin-driven cancers, including CRC (80). Our failed attempts in introducing a complete loss-of-function mutation in the TBX3 gene (this study and reference 29) might indicate that also TBX3, similarly to TBX5, is essential for survival of Wnt-dependent CRC cells. This is important, as, if TBX3 proved to be dispensable for homeostasis as BCL9/9L but required for cancer cell survival as TBX5 or β-catenin, its targeting will constitute the proverbial therapeutic window.

## Methods

TBX3’s role in CRC was investigated using HCT116, DLD1, and SW620 cell lines and HCC patient biopsies. CUT&RUN LoV-U with ICEBERG captured genome-wide TBX3 chromatin association, while CAGE-sequencing in HCT116 cells (control and TBX3-overexpressing) linked regulatory mechanisms to gene expression. CRISPRi knockdown in HCT116 validated direct TBX3 targets, analyzed via RT-PCR. BioID in HEK293T identified TBX3-associated nuclear proteins. Protein–protein and protein–DNA interactions were explored in silico (Alphafold2/3) and experimentally by deleting motifs in TBX3’s DNA-binding domain. TBX3’s role in Wnt transcription was tested via a Top-Flash assay in HEK293T. Extended methods are in Supplementary Information (*SI Appendix*).

## Supplementary Material

Appendix 01 (PDF)

Dataset S01 (XLSX)

Dataset S02 (XLSX)

Dataset S03 (CSV)

Dataset S04 (XLSX)

## Data Availability

CAGE-seq raw; CUT&RUN-LoV-U raw and processed, MS proteomics data have been deposited in ArrayExpress, PRIDE (E-MTAB-13647, E-MTAB-13646, PXD047899) (81–83). All study data are included in the article and/or supporting information. Previously published data were used for this work (https://doi.org/10.1093/nar/gkae180) (41).
