# Supplementary material for: The developmental factor TBX3 engages with the Wnt/β-catenin transcriptional complex in colorectal cancer to regulate metastasis genes
Source: Proc Natl Acad Sci U S A. 2025 May 9;122(19):e2419691122. doi: 10.1073/pnas.2419691122 (PMC12088458; doi:10.1073/pnas.2419691122)
Supplement: Supplementary file 1 — Appendix 01 (PDF) [file pnas.2419691122.sapp.pdf]

PNAS

Supporting Information for
The Developmental Factor TBX3 Engages with the Wnt/β-catenin Transcriptional Complex in Colorectal Cancer to Regulate Metastasis Genes

Amaia Jauregi-Miguel [1,2,#], Simon Söderholm [1,2,3,#], Tamina Weiss [1,2,3,#], Anna Nordin [1,2,3†], Valeria Ghezzi [1,2,†], Salome M. Brütsch [1,2], Pierfrancesco Pagella [1,2,13], Yorick van de Grift [1,2,3], Gianluca Zambanini [1,2,14], Jacopo Ulisse [1,2], Alessandro Mattia [1,2], Ruslan Deviatiiarov [4,5,6], Elena Faustini [1,2], Lavanya Moparthi [1,2], Wenjing Zhong [1,2], Bergthor Björnsson [7], Per Sandström [7], Erik Lundqvist [8], Francisca Lottersberger [1,2], Stefan Koch [1,2], Andreas E. Moor [9], Xiao-Feng Sun [10], Eleonore von Castelmur [1,11], Guojun Sheng [12], Claudio Cantù [1,2,3, Bs,CA]

Affiliations:
[1] Wallenberg Centre for Molecular Medicine, Linköping University, Linköping, Sweden.
[2] Department of Biomedical and Clinical Sciences, Division of Molecular Medicine and Virology, Faculty of Medicine and Health Sciences, Linköping University, Linköping, Sweden.
[3] Science for Life Laboratory – SciLifeLab – Linköping University, Linköping, Sweden
[4] Regulatory Genomics Research Center, Institute of Fundamental Medicine and Biology, Kazan Federal University, Volkova str. 18, Kazan, Tatarstan, 420012, Russian Federation
[5] Endocrinology Research Center, Dmitriya Ul'yanova str. 11, 115478, Russian Federation
[6] Graduate School of Medicine, Juntendo University, Tokyo, Japan
[7] Department of Surgery in Linköping and Department of Biomedicine and Clinical Sciences, Linköping University, Linköping, Sweden.
[8] Department of Surgery, Vrinnevi Hospital, Norrköping, and Department of Biomedicine and Clinical Sciences, Linköping University, Linköping, Sweden
[9] Department of Biosystems Science and Engineering, ETH Zürich, Schanzenstrasse 44, 4056, Basel, Switzerland
[10] Department of Oncology, and Department of Biomedical and Clinical Sciences, Division of Surgery, Orthopedics and Oncology, Faculty of Medicine and Health Sciences, Linköping University, Linköping, Sweden.
[11] Department of Physics, Chemistry and Biology, Division of Chemistry, Faculty of Science and Engineering, Linköping University, Linköping, Sweden
[12] International Research Center for Medical Sciences, Kumamoto University, Kumamoto, Japan
[13] Current address: Department of Physics, Chemistry, and Biology, Division of Biophysics and Bioengineering, Faculty of Science and Engineering, Linköping University, Linköping, Sweden
[14] Current address: Max-Planck-Institut für molekulare Genetik, Berlin, Germany
[#] First authors
[†] Second authors
[Bs] Bluesky @claudiocantu81.bsky.social

Corresponding author: Claudio Cantù, claudio.cantu@liu.se

This PDF file includes:

Supporting Information: Detailed Methods (pp.2-8)
- Cell Culture
- CUT&RUN LoV-U
- CAGE Sequencing
- CRISPR inhibition knock-out
- BioID Proximity Labeling
- Protein Structure Prediction with AlphaFold
- Input Sequences for AlphaFold Predictions
- TOPFlash Luciferase Reporter Assay
- Data availability statement

Figure S1-S3 (pp.9-12)
Legends for Figure S1-S2
Legends for Supplementary File S1-S4 (p.13)
References for SI Appendix (pp.14-15)

Other supporting materials for this manuscript include the following:

Supplementary File S1-S4

Supporting information: Detailed Methods

Materials

Cell culture. HCT116, DLD1, and SW620 human colorectal cancer cells, as well as HEK293T human embryonic kidney cells, were cultured in a 37 °C incubator in 5% CO_2 and 89% humidity. Culture medium used was high glucose Dulbecco's Modified Eagle Medium (Cat. #41965039, Gibco) supplemented with 10 % bovine calf serum (Cat. #1233C, Sigma-Aldrich) and 1X Penicillin-Streptomycin (Cat. #15140148, Gibco).

Tissues. We obtained biopsies (1-4cm2) from primary Hepatocellular Carcinoma (HCC) through resection performed at Linköping University Hospital. Biopsies were transported in phosphate buffered saline (PBS) on ice for subsequent nuclear extraction. The use of biopsies for this study was approved by Etikprövnings Myndigheten (ethical license 2023-06822-01). All patients gave informed written consent before tumor biopsy was performed. Participant were not compensated. In total, we extracted nuclei from two patients with HCC for this study. Biopsies were washed with PBS 3 times and disected in PBS to 1-5mm2 tissue pieces. Tissue fragments were digested in 1mg/ml Collagenase A (Roche #10103586001) dissolved in PBS supplemented with 0.1% BSA for 45 minutes at 37C while shaking. The resulting cell suspension was filtered through a 70 µm cell strainer and centrifuged at 500g for 5 minutes and thoroughly washed with ice-cold PBS supplemented with 0.1% BSA. Nuclear extraction was performed according to Zambanini et al. 2022.

CUT&RUN LoV-U

Protocol. CUT&RUN LoV-U was performed according to Zambanini et al., 2022 (1), doing 25 replicates of TBX3 in HCT116 and 2 replicates each in DLD1 and SW620 cells, plus IgG negative controls. 250,000 cells/sample were harvested using Trypsin-EDTA (Cat. # 25200056, Gibco) for 5 - 10 minutes. Cells were washed in DPBS two times (Cat. #14190094, Thermo Fisher Scientific). Nuclear extraction was performed by three washes in Nuclear Extraction (NE) buffer (HEPES-KOH pH-8.2 [20 mM], KCl [10 mM], Spermidine [0.5 mM], IGEPAL [0.05%], Glycerol [20%], Roche Complete Protease Inhibitor EDTA-Free). After extraction, nuclei were resuspended in 40 µl NE per sample and bound to 10 µl Magnetic ConA Agarose beads equilibrated in binding buffer (HEPES pH 7.5 [20 mM], KCl [10 mM], $CaCl_2$ [1 mM], $MnCl_2$ [1 mM]) as described in Meers and colleagues (2). Bead binding proceeded for 15 min at 4 degrees, then beads were resuspended in 200 µl wash buffer per sample and distributed in PCR tubes. Samples were washed once in 200 µl EDTA wash buffer (wash buffer with EDTA [0.2 mM]), incubating 5 min at room temperature before being resuspended in antibody buffer (wash buffer with antibody 1:100). Incubation happened ON at 4 °C on a rotator. Antibodies used included anti-TBX3 ABIN6265491 (1:100) and anti-rabbit ABIN101961 (1:100). The next morning samples were washed 5 times and resuspended in 200 µl of pAG-MNase buffer (wash buffer with pAG-MNase 120ng/sample) and incubated for 45 min at 4 °C on a rotator. pAG/MNase was a gift from Steven Henikoff (Addgene plasmid #123461; http://n2t.net/addgene:123461; RRID: Addgene_123461) expressed and purified according to (2). Five washes were performed and during the last wash equilibrated 5 min in wet ice. Samples were resuspended in 200 µl ice cold wash buffer with 2 mM $CaCl_2$for digestion of 30 min in wet ice. The digestion buffer was kept and transferred to tubes containing 1.5 µl of 0.5 M EDTA and 1.5 µl of 0.5 M EGTA to inactivate the pAG-MNase. Beads were resuspended in 47 µl of 1X Urea STOP buffer (NaCl [100 mM], EDTA [2 mM], EGTA [2 mM], IGEPAL [0.5%], Urea [8.8 M]) and the DNA was eluted for 1 hr at 4°C for elution. Beads were collected on the magnet and liquid containing DNA was transferred to the PCR tubes and mixed with the digestion buffer. DNA was purified using two successive rounds of bead purifications according to manufacturer's directions, using Mag-Bind TotalPure NGS beads (Cat. #M1327, Omega Bio-Tek) at 2X (200 µl first round and 40 µl second round), final elution was with 20 µl Tris-HCl pH 7.5.

Library Preparation and Sequencing. Library preparation was performed with the KAPA Hyper Prep Kit for Illumina sequencing (Cat. #KK8504, KAPA Biosystems) according to manufacturer's guidelines with modifications. 0.4X volume reactions were used for End repair and A-tailing steps. The thermocycler conditions were 12 °C for 15 min, 37 °C for 15 min and 58 °C for 25 min. Adapter ligation was also performed in 0.4X volume reactions. KAPA Dual Indexed adapters were used at 0.15 µM. DNA purification was performed after ligation with 1.2X volumes of Mag-Bind TotalPure NGS beads. Library amplification steps were performed in 0.5X volume reactions. The cycling conditions were set to: 45 sec initial denaturation at 98 °C, 15 sec denaturation at 98 °C, 10 sec annealing/elongation at 60 °C, 1 min final extension at 72 °C, hold at 4 °C. Libraries were amplified 13 cycles. DNA purification was performed with 1.2X beads. Libraries were size-selected using the E-Gel EX 2% agarose gel (Cat. # G402022, Invitrogen) and the E-Gel Power Snap Electrophoresis System (Invitrogen), selecting for

fragments 150 - 500 bp. DNA from the gel was purified using the QIAquick Gel Extraction Kit (Cat. #28706X4, Qiagen) according to the manufacturer's instructions. Libraries were quantified using Qubit (Thermo Scientific)'s high sensitivity DNA kit (Cat #Q32854, Thermo Scientific). Libraries were sequenced with 36 bp pair-end reads on the NextSeq 550 (Illumina) using the Illumina NextSeq 500/550 High Output Kit v2.5 (75 cycles) (Cat. #20024906, Illumina).

Data Processing and ICEBERG. Trimming was performed with bbmap bbduk (version 38.18) (3) to remove adapters, known artifacts, and repeats of AT, TA, or poly G/C. Alignment was done to hg38 with bowtie2 (version 2.4.5) (4), settings: –local –very-sensitive-local –no-unal –no-mixed -no-discordant –phred33 –dovetail -I 0 -X 500. SAMtools (version 1.11) (5) was used to fix improperly paired mates and for deduplication. Bam files were filtered with the CUT&RUN hg38 blacklist (suspect list) (6) using BEDTools (version 2.30.0) (7). BEDTools genomecov on pair-end mode was used to create bedgraphs, bedgraphs were visualized in IGV (8). Data was processed with the ICEBERG pipeline according to Nordin et al., 2023a, to generate the TBX3 ICEBERG dataset. Briefly, individual replicates were peak called with MACS2 (version 2.2.6) (10) with the options -f BAMPE --keep-dup all and -q 5e-2 against the control. The maximum number of usable fragments was determined by the smallest replicate, and each replicate was shuffled and downsampled to this depth 3X, then merged into aggregates. Each were separately peak called with MACS2 as described above. Between each replicate addition, peaks were called to generate the ICEBERG build curve, which was modeled by fitting polynomial regressions of increasing order (up to 5th) in R (version 4.2.2, lm function), selecting that which had the highest R2 value. Peaks called in at least 2 of the 3 aggregates were determined and filtered to remove peaks not called in at least one individual replicate at MACS2 -p 1e-2, to finally generate the final set of ICEBERG peaks for TBX3.

Downstream Analyses and Integration with Published Data. Signal intensity plots and profiles were generated using ngs.plot (version 2.63) (11). Genomic region annotation was done with HOMER (version 4.11) (5) annotatePeaks on default settings, and motif/consensus analysis was also done with HOMER with the -size given parameter. Annotation to genes was done using GREAT (version 4.0.4) (12) on default settings. Gene ontology and KEGG pathway analysis was done with ShinyGO (13). Intervene (version 0.6.4) (14) was used to create Venn diagrams.

CUT&RUN datasets of β-catenin were downloaded from Nordin et al., 2023a and overlapped with TBX3 ICEBERG peaks using bedtools or Intervene. HiChIP of H3K27ac in HCT116 data, performed by Chen and colleagues (15) was downloaded from GEO. Loops were filtered as described in Nordin et. al., and then filtered for those overlapping TBX3 peaks using BEDtools pairtobed. Bigwig files mapped to hg38 were downloaded for H3K27ac conducted in HCT116 (16) by the ENCODE consortium, and were used for visualization. Peak and annotated genes can be found in Supplementary File 1.

HEPG2 ChIP-seq tracks. Following publicly available transcription factor Chromatin-Immuno-Precipitation (ChIP) sequencing tracks where downloaded from ENCODE (https://www.encodeproject.org/) as bigwig files: ENCFF287XCG_TBX3_HEPG2; ENCFF397PQZ_TBX3HEPG2; ENCFF371KMF_TCF7HEPG2; and ENCFF406WNX_TCF7L2_HEPG2.

CAGE Sequencing

To investigate the transcriptional consequences upon TBX3 overexpression, we performed Cap Analysis of Gene Expression followed by next generation sequencing (CAGE-seq). This method allows genome-wide transcriptional profiling of genes as well as accurate identification of transcriptional start sites (TSSs) and their corresponding promoter regions.

Library Preparation and Sequencing. HCT116 were transfected with either TBX3-3xFlag (donated by Peter J. Hurling) or the empty vector control, each in three biological replicates. Total RNA was isolated by using a standard TRIzol-based method (Cat#15596018, Thermo Scientific). The quality of the extracted RNA was tested on the Agilent 2100 Bioanalyzer. Each sample consisted of 5 µg of initial total RNA. Library preparation, including cap-trapping step and polyadenylation, was conducted according to a standard CAGE protocol (17). Briefly, RNA was reverse transcribed using random primers, followed by biotinylation of the RNA cap and 3' ends. Single-stranded, non-hybridized RNAs were digested with RNase leaving a 5' cDNA that was captured by streptavidin beads. Sequencing was performed following the manufacturer instructions.

Data Analysis. Quality of the obtained sequencing reads was assessed using FastQC (Andrews S. (2010; https://www.bioinformatics.babraham.ac.uk/projects/fastqc/). Bad quality (phred score < 33) and N containing reads were filtered out by fastx_trimmer (FASTX Toolkit, version 0.0.14) and removeN (Moirai), respectively. Reads matching ribosomal DNA (U13369.1.fa) were removed with RNAdust (version 1.06) and adapter sequence contaminants were removed with Trimmomatic (18), version 0.38, with the following settings: SE -phred33 LEADING:30 TRAILING:25 SLIDINGWINDOW:5:30

MINLEN:30. Trimmed and filtered reads were aligned to the UCSC human reference genome hg38 using the Burrows-Wheeler Aligner (BWA) (19), version 0.7.10-r789, with BWA-backtrack algorithm and the following settings: -n 0.02 -o 1 -e -1 -l 5 -d 10 -l 32 -k 2 -m 2000000 -M 3 -O 11 -E 4 -R 30 -q 0. Unmapped reads were re-aligned with Hisat2 (20), version 2.1.0. Obtained alignment files in SAM format were converted to the binary BAM format, coordinate sorted and indexed with SAMtools (21), version 1.11-5-g0920974. Next, CAGE alignments were processed by PromoterPipeline (22) in order to get CTSS (CAGE transcription start sites) and CAGE peaks, which were associated with nearby Gencode v38 gene models by using ChIPseeker v1.32.0 package for R. CAGE peaks located within known promoter regions (2kbp) were used as training set in TSSClassifier (23) to predict whether sequence composition of remaining peaks is similar to promoters. CAGE peaks located in known promoters or classified as promoter associated were used for gene expression analysis and masked for enhancers calling (24). CAGE promoters to enhancers associations defined for pairs located within 500kbp and correlation test p.value < 0.05. CAGE expression profiles, promoters, enhancers, and associations are available through Zenbu browser: https://fantom.gsc.riken.jp/zenbu/gLyphs/#config=TBX3%20CAGE. CAGE regions, annotated genes, counts and differential expression can be found in Supplementary File 2.

Integration of CAGE-seq and CUT&RUN. Genomic coordinates of enriched CAGE regions were input into a bed file and overlapped with filtered H3K27ac mediated chromatin loops, keeping loops with at least one anchor in a DEG region. Next, these loops were overlapped with TBX3 and then β-catenin ICEBERG peak sets. The corresponding CAGE/H3K27ac/TBX3/β-catenin loops were annotated to genes using GREAT, using coordinates of both anchors of each loop. Pathway enrichment was performed as described above. The results can be found in Supplementary File 3.

CRISPR inhibition knock-down

Cloning. Guides targeting within 50bp upstream or downstream the transcription start site of TBX3 were taken from the Dolcetto library (25) (guide 1&2) or designed using CHOPCHOP webtool (26) (guide 3; https://chopchop.cbu.uib.no/) and shown in Table 1. A scrambled, non-targeting (NT) control was included. Guides were annealed, phosphorylated and ligated into the LentiV2(BB)-EFIa-KRAB-dCas9-P2A-BlastR (Addgene#118154) upon Esp3I digestion following the Zhang lab protocol. Successful integration of the guides into the vector was validated via colony PCR.

# pair	strand	oligo target sequence
hTBX3_sgRNA1	+	5'CACCGATTCTAGAGGCGGCGGAGGG'3
	-	3'CTAAGATCTCCGCCGCCTCCCCAAA'5
hTBX3_sgRNA2	+	5'CACCGAGACGCCCGGTGAATTCTAG'3
	-	3'CTCTGCGGGCCACTTAAGATCCAAA'5
hTBX3_sgRNA3	+	5'CACCGGAATTCACCGGGCGTCTGCT'3
	-	3'CCTTAAGTGGCCCGCAGACGACAAA'5
NTscramble_sgRNA	+	5'CACCGGTATTACTGATATTGGTGGG'3
	-	3'CCATAATGACTATAACCACCCCAAA'5

Table 1. CRISPRi guide sequences with BsmbI overhang

Virus production and infections. Lentiviral particle were produced in HEK293T (passage<10). Cells were transfected 6h after seeding 5 million cells per 10cm tissue culture dish using a mix of calcium phosphate, the packaging plasmids ENV (3 µg); RREI (5 µg); and REV (2.5 µg), and either a pool of the three CRISPRi sgTBX3 vectors (3 µg each) or the CRISPRi sgNT control vector (9 µg). Starting from 48h post transfection, the virus containing media was collected every 12h and filtered. After adding polybrene to a final concentration of 4 µg/mL, the virus containing media was used for 4 subsequent infections of HCT116 cells. 12h after the last infection, cells were lysed with Trizol (Qiagen #79306).

Reverse-Transcription PCR. RNA was extracted from the lysates following the manufacturer's instructions. 2000ng RNA per sample was reversed transcribed using the High Capacity cDNA Reverse Transcription Kit (Thermo Fisher/appliedbiosystems #4368813). 3 µL of 1:4 diluted cDNA was mixed with 2uL primer [2 µM] (Table 2) and 5 µL SsoAdvanced Universal SYBR Green Supermix (BioRad #172574) and analyzed in 40 cycles with the BioRad CFX96 Real-Time System. Relative expression levels were normalized to intrasample GAPDH RNA levels and visualized as fold change over the non-targeting control, according to the ddCT method. An independent t-test was calculated comparing the means of the knock-down and NT-control conditions for each target.

Target		Primer sequence 5'->3'	Target		Primer sequence 5'->3'
hTBX3	Fwd	GTGTCTCGGGCCTGGATTC	hEZR	Fwd	ACCAATCAATGTCCGAGTTACC
hTBX3	Rvs	ACGTGTAGGGGTAAGGGAACA	hEZR	Rvs	GCCGATAGTCTTTACCACCTGA
hAXIN2	Fwd	CTGGCTTTGGTGAACTGTTG	hFN1	Fwd	TGGGACTGTACCTGCATCGGGG
hAXIN2	Rvs	AGTTGCTCACAGCCAAGACA	hFN1	Rvs	GACTGACCCCCTTCATGGCAGC
hCDH1	Fwd	GAGCTACACGTTCACGGTGCCC	hSNAIL1	Fwd	AGTGGTTCTTCTGCGCTACT
hCDH1	Rvs	GGCTGTCCTTTGTCGACCGGTG	hSNAIL1	Rvs	GTAGGGCTGCTGGAAGGTAA
hEGFR	Fwd	CGGACTGAAGGAGCTGCCCATG			
hEGRF	Rvs	AGGGCAGGGTTGTTGCTGAACC			

Table 2. RT-PCR primer sequences

BioID Proximity Labeling

BirA Plasmid Construction. The BioID fused-protein construct was prepared by cloning the TBX3 gene sequence into a BioID pCS2 flag plasmid in-frame with the biotin ligase BirA at the C-terminal. In brief, the TBX3 coding sequence was PCR-amplified using a gradient-based PCR program (98°C 1 min – 98°C 30 sec – 62°C 30 sec – 72°C 1 min – GOTO Step 2 7x – 98°C 30 sec – 72°C 2 min – GOTO step 6 30x – 72°C 5 min – hold 4°C). PCR product was purified using MicroElute® Cycle-Pure-Kit (Omega Bio-Tek) and ligated into the BioID plasmid using type II restriction enzymes BamHI and XhoI (New England BioLabs) and T4 DNA ligase. 3 µL of ligation product was used for transformation of competent DH5α cells (Thermo Scientific) and plated on ampicillin-selective agar plates. 4 colonies were selected for further culturing before cell lysis and plasmid DNA purification using MiniPrep Kit (Thermo Scientific). To generate the NPF motif deleted TBX3-BirA construct, the NEB Q5 Site-directed Mutagenesis Kit (New England BioLabs, USA) was used, following the manufacturer's instructions. All constructs were confirmed by partial sequencing (Eurofins Genomics).

Cell Transfection, Pull-down and Protein Sample Preparation. HEK293T cells were transfected at 30-40% confluency in T75 flasks with 11.25 µg of C-terminal BirA-TBX3 plasmids (empty vector, TBX3 wild-type, TBX3DNPF, and TBX3DRRM). Media was refreshed 24 hours post-seeding. At 48 hours post-transfection, cells were treated with 50 µM biotin and either 10 nM LGK or 10 µM CHIR. At 72 hours, approximately 3*10^6 cells per condition were harvested. Cells were detached using trypsin, washed twice with PBS, and kept on ice. For nuclear extraction, cells were pelleted by centrifugation at 800g for 5 minutes and resuspended in 300 µL nuclear extraction buffer (10 mM HEPES pH 7.9, 10 mM KCl, 0.1 mM EDTA, 0.1 mM EGTA, 1 mM DTT, and 1 mM PMSF). After a 15-minute incubation on ice, the cells were vortexed for 10 seconds and centrifuged at 10,000g for 30 seconds. The nuclear pellet was resuspended in 100 µL of cold nuclear lysis buffer (20 mM HEPES pH 7.9, 0.4 M NaCl, 1 mM EDTA, 1 mM EGTA, 1 mM DTT, and 1 mM PMSF), shaken vigorously for 15 minutes at 4°C, and centrifuged at maximum speed for 15 minutes at 4°C.

For the pull-down assay, nuclear protein lysates were sonicated (40 mA, 30-second on/off cycles for 5 minutes). Concurrently, 60 µL of Streptavidin Sepharose High Performance beads (Cytiva #17-5113-01) were washed in low binding tubes with 600 µL of nuclear lysis buffer (centrifuged for 2 minutes at 2000g) and resuspended in 60 µL of lysis buffer. Samples (total volume approximately 200 µL) were added to the beads and incubated for 3 hours at 4°C with end-over-end rotation. Subsequently, the beads were centrifuged for 2 minutes at 2000g and washed three times with 1 mL of 50 mM ammonium bicarbonate. Beads were resuspended in 200 µL of 50 mM ammonium bicarbonate containing 0.4 µg of Pierce MS Grade Trypsin Protease (Thermo Scientific, Cat. #90057). Tubes were sealed and incubated for 1 hour at 37°C. Digested proteins were released in three sequential steps with 50 mM ammonium bicarbonate by resuspending the beads and collecting the supernatant (200 µL, 2x 100 µL). The 400 µL pooled supernatant was centrifuged for 10 minutes at 14,000 rpm, and the supernatant was transferred to a new tube. Fresh 100 mM DTT was added to a final concentration of 5 mM and incubated for 30 minutes at 37°C. Fresh 100 mM iodoacetamide was added to a final concentration of 12.5 mM and incubated for 45 minutes at room temperature. An additional 0.5 µg of trypsin was added, and the samples were incubated for digestion overnight at 37°C on a rotator. Samples were desalted using Pierce C18 columns (Thermo Fisher, Cat. #89870). Briefly, samples were dried in a speed vacuum and resuspended in 40 µL of resuspension solution (0.5% TFA, 5% ACN). Columns were placed in a collection tube and activated twice with 200 µL of 50% ACN (centrifuged for 1 minute at 1500 rpm). After two rounds of 200 µL equilibration solution (0.5% TFA, 5% ACN, centrifuged for 1 minute at 1500 rpm), columns were placed in a fresh tube, and samples (40 µL) were loaded, incubated, and centrifuged. Sample-bound columns were washed twice with 200 µL of 0.5% TFA and 5% ACN. Columns were then transferred to a new tube, and the sample was eluted with 20 µL of elution buffer

(70% ACN, 0.03% FA) in three rounds (final volume 60 µL). Samples were completely dried in a speed vacuum at room temperature and stored at -20°C until further processing.
Mass Spectrometry Data Acquisition. 10 µL of sample was transferred to Polypropylene Snap Top Microvials (6ERV11-08PPC, Thermo Fisher Scientific) and analyzed by mass spectrometry, using an Easy nano LC 1200 system interfaced with a nanoEasy spray ion source (Thermo Fisher Scientific) connected Q Exactive HF Hybrid Quadrupole-Orbitrap Mass Spectrometer (Thermo Fisher Scientific). The peptides were loaded on a pre-column (Acclaim PepMap 100, 75 µm x 2 cm, Thermo FisherScientific) and the chromatographic separation was performed using an EASY-Spray C18 reversed-phase nano LC column (PepMap RSLC C18, 2 µm, 100A 75 µm x 25 cm, Thermo Fisher Scientific). A linear gradient of 6-30% buffer B (0.1% formic acid in acetonitrile) against buffer A (0.1% formic acid in water) during 65 min and 100% buffer B against buffer A till 90 min was carried out with a constant flow rate of 300 nL/min. Separated peptides were electrosprayed and analyzed using a Q-Exactive HF mass spectrometer (Thermo Fisher Scientific), operated in positive polarity in a data-dependent mode. Full scans were performed at 120,000 resolutions at a range of 380–1400 m/z. The top 15 most intense multiple charged ions were isolated (1.2 m/z isolation window) and fragmented at a resolution of 30,000 with a dynamic exclusion of 30.0 s.
Data Analysis. Raw mass spectrometry data was analyzed for peptide identification and quantification with Proteome Discovererer 2.5 (Thermo Fisher Scientific), using the SequestHT search engine against the Homo sapiens UniProt database (UP000005640; 852,685 entries) in which Streptavidine and BirA also had been added. Cysteine carbamidomethylation was used as static modification and methionine oxidation as dynamic modification for both identification and quantification. A maximum of 2 trypsin cleavages were allowed, and the precursor and fragment mass tolerance were 15 ppm and 0.1 Da, respectively. Peptides with false discovery rate (FDR) of less than 0.01 were considered significant, and the minimum peptide length was set to 5.
Results were filtered to remove common contaminants, including serum albumin, keratins, tubulins, heat shock proteins, and streptavidin. Processed mass spectrometry data were further analyzed to determine enrichment of proteins in treatment samples over controls using SAINTexpress (27), an implementation of the significance analysis of interactome (SAINT) algorithm (28). Total spectral counts were used as input. A SAINT score > 0.6 and an estimated Bayesian false discovery rate (BFDR) < 0.05 were used as thresholds for significance. Overlap analysis and plotting of data were performed with the R programming language (R Core Team, 2017), in Rstudio (Rstudio Team, 2015). The mass spec results of all tested conditions can be found in Supplementary File 4. Note that these are the raw data, before any filtering of contaminants and prior to SAINT analysis.

Protein Structure Prediction with AlphFold
The decision to exclusively generate models for the highly structured T-box domain was motivated by the presence of the NPF and the RRM motifs, while also the remainder of the protein exhibited predominantly unstructured characteristics.
AlphaFold2. Structural predictions were done with ColabFold (v.1.5.3), a software package that offers an integrated protein prediction solution as a web-based interface (29). This tool integrates rapid homology searches, employing many-against-many sequence searching (MMseqS2) against the databases UniRef100, PDB100, and environmental sequence database, in conjunction with AlphaFold2 and RoseTTAFold methodologies (30–32).The predictions were executed with the standard settings recommended by the developers. In brief, the input sequences (see below) were provided to the query_sequence command. Num_relax was set to 0. Template mode was set to none. When predicting complexes consisting of multiple proteins or domains the sequences of all subunits were concatenated using a colon (':'). MMseqS2_uniref_env, in unpaired_paired mode, was selected for multiple sequence alignment. For modeling, alphafold2_multimer_v3 was chosen for protein complexes, while alphafold2_ptm was selected for protein structure modeling. The default setting of 5 models with one single seed value was used. Recommended settings for both models were used to predict protein structure. Protein structures were checked for consistency across five generated models. The results presented in the figures pertain solely to the top-ranking model (rank 1 out of 5). For monomer predictions the top-ranking model was determined by the highest average local distance difference test (LDDT) score—an indicator of model accuracy estimation. For monomer predictions standard T4 TPU's with 12gb RAM were used. The settings to generate Figure 6C were adapted from (33).
AlphaFold3. To predict protein-DNA interaction (Figure 6D), AlphaFold3 (34) model was used running on the DeepMind prediction server (https://alphafoldserver.com). To design the input DNA probes, the consensus sequences (MA0768.1, MA0099.4) of two top enriched DNA motifs (LEF1, Jun/AP1) of the TBX3 C&R ICEBERG in HCT116 were downloaded from JASPAR (https://jaspar.elixir.no) and used to scan the chromosomal locations of the MYC or WNT9A peak coordinates using the MEME tool FIMO

(v.5.5.7, https://meme-suite.org/meme/tools/fimo). Both searches resulted in a single highly significant hit located in the peak summit. Subsequently, the consensus sequences, including the surrounding endogenous 40 nucleotides, were chosen as DNA-input sequence. The sequences to model the interaction of TBX3 with its TBX DNA consensus were obtained from Coll et al. (35). The AF3 predictions were executed with the standard settings recommended by the developers. In brief, the input sequences (see below) were inserted specifying the molecule type and number of copies. The default setting of 5 models were used with the seed option set to 'auto'. Protein structure/ interactions were checked for consistency across the five generated models. The results presented in the figures pertain solely to the top-ranking model (rank 0 out of 4). To visualize the confidence scores, prediction alignment error and contact probability parts of the the publicly available python script AF3_score_visualizer.ipynb (https://colab.research.google.com/drive/1YB2378jqsZMNkVPonF1Mxrp-jn188w16?usp=sharing#scrollTo=erV7Loh-cssf) was used. Visualization of pdb files was done with Mol* (36) of RCSB PDB.

Input Sequences for AlphaFold Predictions

Sequences for Figure 6.B (PDB 1H6F dimer)
>1H6F_1|Chains A, B|T-BOX TRANSCRIPTION FACTOR TBX3|HOMO SAPIENS (9606)
MKDDPKVHLEAKELWDQFHKRGTEMVITKSGRRMFPPFKVRCSGLDKKAKYILLMDIIAADDCRYKF
HNSRWMVAGKADPEMPKRMYIHPDSPATGEQWMSKVVTFHKLKLTNNISDKHGFTILNSMHKYQPR
FHIVRANDILKLPYSTFRTYLFPETEFIAVTAYQNDKITQLKIDNNPFAKGFRDTGNGRR
>1H6F_2|Chains C, D|5'-D(*TP*AP*AP*TP*TP*TP*CP*AP*CP*AP*CP*CP*TP*
AP*GP*GP*TP*GP*TP*GP*AP*AP*AP*T)-3' (32630)
TAATTTCACACCTAGGTGTGAAAT

Sequences for Figure 6.C (AlphaFold2)
Left: TBX3 (UniProt O15119) T-Box domain wild-type +20aa
KTMEPEEEVEDDPKVHLEAKELWDQFHKRGTEMVITKSGRRMFPPFKVRCSGLDKKAKYILLMDIIAA
DDCRYKFHNSRWMVAGKADPEMPKRMYIHPDSPATGEQWMSKVVTFHKLKLTNNISDKHGFTLAFP
SDHATWQGNYSFGTQTILNSMHKYQPRFHIVRANDILKLPYSTFRTYLFPETEFIAVTAYQNDKITQLKI
DNNPFAKGFRDTGNGRREKRKQLTLQS
Middle: TBX3 (UniProt O15119) T-Box domain - NPF deleted +20aa
KTMEPEEEVEDDPKVHLEAKELWDQFHKRGTEMVITKSGRRMFPPFKVRCSGLDKKAKYILLMDIIAA
DDCRYKFHNSRWMVAGKADPEMPKRMYIHPDSPATGEQWMSKVVTFHKLKLTNNISDKHGFTLAFP
SDHATWQGNYSFGTQTILNSMHKYQPRFHIVRANDILKLPYSTFRTYLFPETEFIAVTAYQNDKITQLKI
DNAKGFRDTGNGRREKRKQLTLQS
Right: TBX3 (UniProt O15119) T-Box domain – RRM deleted +20aa
KTMEPEEEVEDDPKVHLEAKELWDQFHKRGTEMVITKSGFPPFKVRCSGLDKKAKYILLMDIIAADDC
RYKFHNSRWMVAGKADPEMPKRMYIHPDSPATGEQWMSKVVTFHKLKLTNNISDKHGFTLAFPSDH
ATWQGNYSFGTQTILNSMHKYQPRFHIVRANDILKLPYSTFRTYLFPETEFIAVTAYQNDKITQLKIDNN
PFAKGFRDTGNGRREKRKQLTLQS

Sequences for Figure 6.D (AlphaFold3)
Top: TBX3 motif (MA1566.1) in 24bp DNA from Coll et al.
DNA 1: TAATTTCACACCTAGGTGTGAAAT
DNA 2: ATTTCACACCTAGGTGTGAAATTA
Protein 1+2 (T-box):
MKDDPKVHLEAKELWDQFHKRGTEMVITKSGRRMFPPFKVRCSGLDKKAKYILLMDIIAADDCRYKF
HNSRWMVAGKADPEMPKRMYIHPDSPATGEQWMSKVVTFHKLKLTNNISDKHGFTILNSMHKYQPR
FHIVRANDILKLPYSTFRTYLFPETEFIAVTAYQNDKITQLKIDNNPFAKGFRDTGNGRR
Middle: LEF1 motif (MA0768.1) in *MYC* regulatory region peak summit (Hg38 chr8:127742365-127743603) with additional nucleotides flanking:
DNA 1: ATGCAATCCACAGAAGTATAGTAGTTCAAAGGGTTACAAAAGCAAGGCGCTCTT
DNA 2: AAGAGCGCCTTGCTTTTGTAACCCTTTGAACTACTATACTTCTGTGGATTGCAT
Protein 1+2 (T-box): see above (top)
Bottom: Jun/AP1 motif (MA0099.4) in *WNT9A* regulatory region peak summit (Hg38) with additional nucleotides flanking:
DNA 1: ACTCTGTGGTGAACTGAGGCTGTGACTCAGGCTCTGTATAGTGAACTGGGGCTG
DNA 2: CAGCCCCAGTTCACTATACAGAGCCTGAGTCACAGCCTCAGTTCACCACAGAGT
Protein 1+2 (T-box): see above (top)

Protein Conservation Prediction. Protein conservation estimates were done with CoservFold (V.1, 2023) (ConservFold.ipynb – Colaboratory (google.com)) (Graham C, Stansfeld P and Rodrigues C, Conservation-Colab: Conservation to 3D structure, Github, 10.5281/zenodo.10062701, 2023). This Google Collaboratory based platform generates a multiple sequence alignment file with MMSEQ2 (99). Subsequent run of Weblogo3 (105) calculates entropy scores from the sequence similarities. The predictions were executed with the standard settings recommended by the developers.

TOPFlash Luciferase Reporter Assay

To conduct reporter assays, HEK293T cells were seeded in a 96-well plate overnight and co-transfected with 50 ng of firefly reporter, 5 ng of renilla control, and 10 ng of the plasmid of interest in each well. M50 Super 8x TOPFlash (SuperTOPFlash is found at Addgene, Plasmid #12456) and M51 Super 8x FOPFlash (Addgene #12457) as used before (37, 38). In the indicated samples cells were treated with control or CHIR99021 (1 µM, Cat. #SML1046, Sigma Aldrich) 6 h after transfection. The dual luciferase assay was performed with some modifications as previously described (39). After overnight incubation, cells were lysed in passive lysis buffer (25 mM Tris, 2 mM DTT, 2 mM EDTA, 10% (v/v) glycerol, 1% (v/v) Triton X-100, pH 7.8) and agitated for 10 min. The lysates were then transferred to a flat-bottomed 96-well luminescence assay plate. Firefly luciferase buffer (200 µM D-luciferin in 200 mM Tris-HCl, 15 mM $MgSO_4$, 100 µM EDTA, 1 mM ATP, 25 mM DTT, pH 8.0) was added to each well and the plate was incubated for 2 min at room temperature. The luciferase activity was measured using a SpectraMax iD3 Multi-Mode Microplate Reader (Molecular Devices). Subsequently, Renilla luciferase buffer (4 µM coelenterazine-h in 500 mM NaCl, 500 mM Na_2SO_4, 10 mM NaOAc, 15 mM EDTA, 25 mM sodium pyrophosphate, 50 µM phenyl-benzothiazole, pH 5.0) was added to the plate, and luminescence was immediately measured. The data were normalized to the Renilla control values, performed in triplicate, and the Top/Renilla ratio was used as an indicator of β-catenin-driven transcription.

Statistical Analysis. The R programing language was used for statistical analyses. Data were visualized in box and whiskers plot. Student's t-test was used to analyze the pairwise differences between groups and $p<0.05$ was considered statistically significant. All experiments were performed at least three times, and all samples were analyzed in six replicates unless otherwise stated.

Data Availability Statement

Cage-seq raw and processed data have been deposited to ArrayExpress and can be accessed via accession number E-MTAB-13647. CUT&RUN-LoV-U raw and processed data have been deposited to ArrayExpress and is available via the accession number E-MTAB-13646. The mass spectrometry proteomics data have been deposited to the ProteomeXchange Consortium via the PRIDE partner repository (40) with the dataset identifier PXD047899.

Supplementary Figure 1

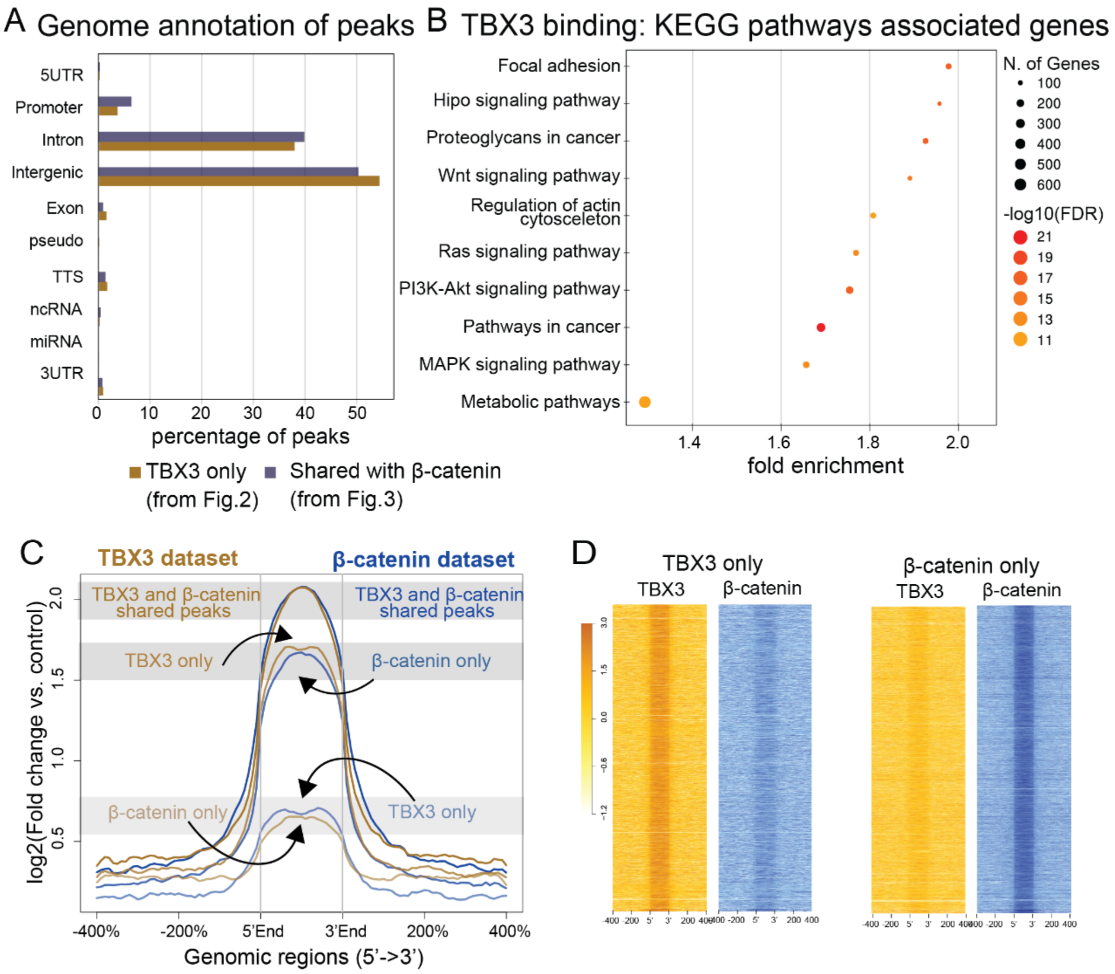

Figure S1. Supporting information for Figure 2&3 CUT&RUN ICEBERGs in HCT116

(A) Genome annotation of peaks called in TBX3 and β-catenin CUT&RUN ICEBERGs.

(B) KEGG pathway enrichment of the set of TBX3 peak associated genes (gene annotation done with GREAT), showing enrichment for many cancer-related signaling pathways, including Wnt and MAPK.

(C)Signal profiles of TBX3 and β-catenin CUT&RUN signal in TBX3 only peaks, shared peaks, and β-catenin only peaks. Brown toned colors represent peaks in the TBX3 dataset, blue toned colors represent peaks in the b-catenin data-set.

(D) Signal intensity plots of TBX3 and β-catenin CUT&RUN signal in TBX3 only peaks (left) and β-catenin only peaks (right).

Supplementary Figure 2

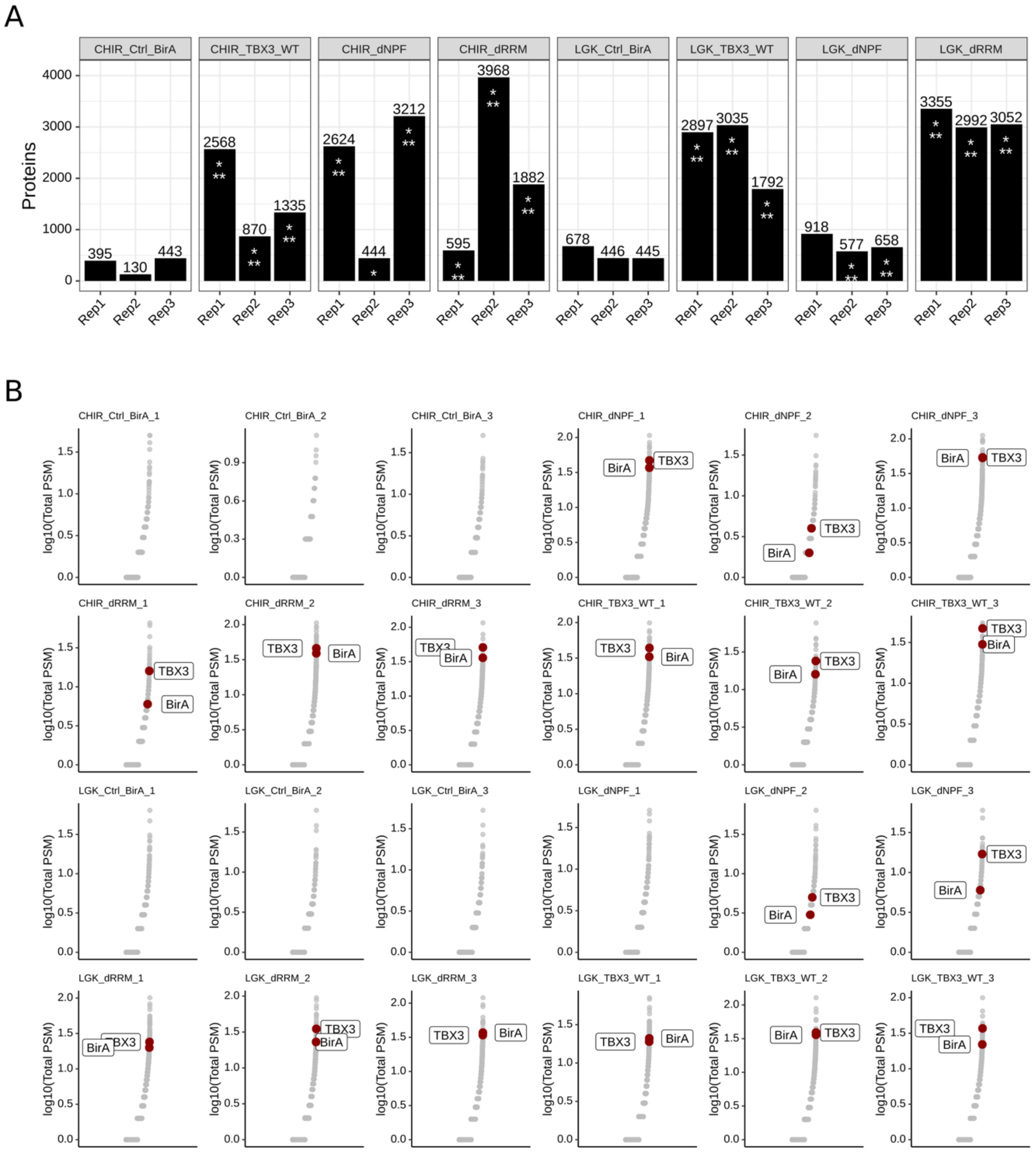

Figure S2. Supporting data for the TBX3 proximity labeling.

(A) Barplots showing total number of protein hits obtained by mass spectrometry. Results have been filtered to remove contaminants, including serum albumin, keratins, tubulins, heat shock proteins, and streptavidin. Single asterisk (*) denotes detection of TBX3, while double asterisk (**) denotes detection of BirA.

(B) Dotplots showing detected proteins ranked by log10 total peptide-spectrum matches (y-axis) for each separate sample replicate. The position of TBX3 and BirA has been highlighted in darkred. Results have been filtered to remove contaminants, including serum albumin, keratins, tubulins, heat shock proteins, and streptavidin.

Supplementary Figure 3

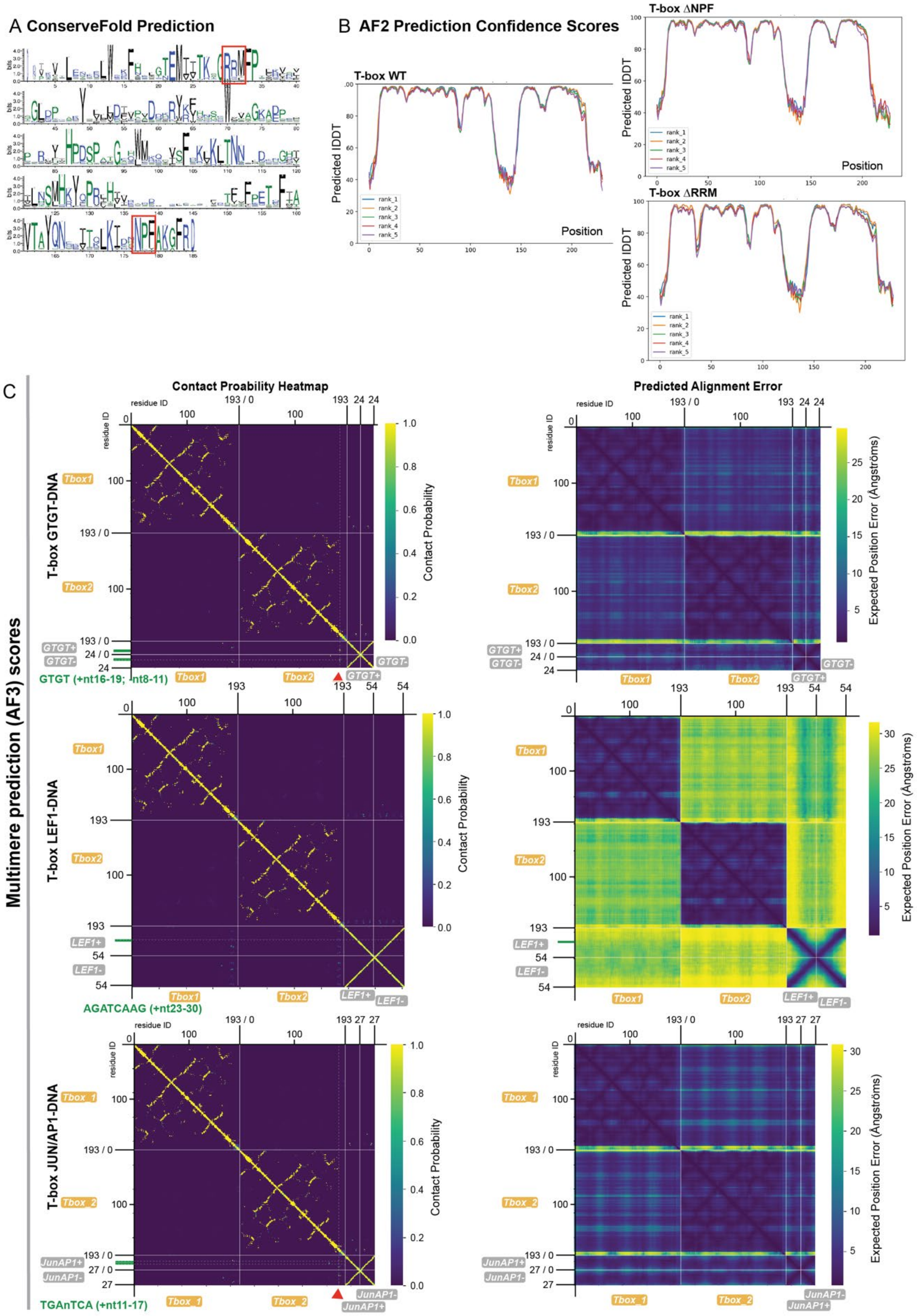

Figure S3. Supporting information for AlphaFold structure prediction.

(A) Left: WebLogo of the ConservFold prediction analysis for the human TBX3T-box domain. Shown are residue 0-185 of the T-box domain (x-axis) and their conservation score informational contant (y-axis in bits)

(B) Confidence scores of the AlphaFold2 structural prediction of the sequence indicated above each plot. Y-axis: pIDDT for local Distance Difference Test, a per-residue measure of confidence. X-axis: residue position within the protein domain. Shown are model rank 1-5 with no abarrent differences.

(C) AlphaFold3 prediction's contact probability map and predicted alignment error calculated for the structures shown in Figure 6D. Green marks indicate location of the respective DNA consensus sequence; red triangle indicates the location of the NPF motif.

Legend for Supplementary Files

Supplementary File S1 (separate file). CUT&RUN data.
Sheet 1: GREAT associated Genes in TBX3 ICEBERG peaks

Sheet 2: Peak overlap of TBX3 and bCat ICEBERG

Sheet 3: GREAT associated genes in sheet 2 overlap peaks

Sheet 4: CAGE DEGs (non-stringent filtering q<0.001, log2FC+/- 0.1)

Sheet 5: "CAGE_loops_Bcat_TBX3" Integrated data: H3K27ac HiChIP loops with TBX3 and bCat CUT&RUN peaks.

A-C: Chr, start, end for the first anchor of the H3K27ac HiChIP loop,

D-F: Chr, start, end for the second anchor of the H3K27ac HiChIP loop

G: H3K27ac HiChIP loop strength

H: H3K27ac HiChIP loop length

I-K: Chr, start, end for the Bcat/TBX3 CUT&RUN peak intersect that the H3K27ac HiChIP overlaps with.

Supplementary File S2 (separate file). TBX3 overexpression in HCT116 CAGEseq results: regions, annotations, counts, differentially expressed genes

Supplementary File S3 (separate file). KEGG enrichment results, list of genes resulting from CAGE data integration with CUT&RUN and HiChIP.

Supplementary File S4 (separate file). Table of BioID results for all tested conditions. Please note that this table includes all proteins obtained from mass spectrometry before filtering of contaminants and prior to SAINT analysis.

SI Appendix References

1. G. Zambanini, A. Nordin, M. Jonasson, P. Pagella, C. Cantù, A new CUT&RUN low volume-urea (LoV-U) protocol optimized for transcriptional co-factors uncovers Wnt/β-catenin tissue-specific genomic targets. *Dev.* **149** (2022).
2. M. P. Meers, T. D. Bryson, J. G. Henikoff, S. Henikoff, Improved CUT&RUN chromatin profiling tools. *Elife* **8**, 1–16 (2019).
3. B. Bushnell, J. Rood, E. Singer, BBMerge – Accurate paired shotgun read merging via overlap. 1–15 (2017).
4. B. Langmead, S. L. Salzberg, Fast gapped-read alignment with Bowtie 2. *Nat. Methods* **9**, 357–359 (2012).
5. H. Li, *et al.*, The Sequence Alignment/Map format and SAMtools. *Bioinformatics* **25**, 2078–2079 (2009).
6. A. Nordin, G. Zambanini, P. Pagella, C. Cantù, The CUT&RUN suspect list of problematic regions of the genome. *Genome Biol.* **24**, 1–18 (2023).
7. A. R. Quinlan, I. M. Hall, BEDTools: A flexible suite of utilities for comparing genomic features. *Bioinformatics* **26**, 841–842 (2010).
8. J. T. Robinson, *et al.*, Integrative genomics viewer. *Nat. Biotechnol.* **29**, 24–26 (2011).
9. A. Nordin, P. Pagella, G. Zambanini, C. Cantù, Exhaustive identification of genome-wide binding events of transcriptional regulators with ICEBERG. *bioRxiv* (2023). https://doi.org/10.1101/2023.06.29.547050.
10. Y. Zhang, *et al.*, Model-based analysis of ChIP-Seq (MACS). *Genome Biol.* **9** (2008).
11. L. Shen, N. Shao, X. Liu, E. Nestler, ngs . plot : Quick mining and visualization of next-generation sequencing data by integrating genomic databases. 1–14 (2014).
12. C. Y. McLean, *et al.*, GREAT improves functional interpretation of cis-regulatory regions. *Nat. Biotechnol.* **28**, 495–501 (2010).
13. S. X. Ge, D. Jung, D. Jung, R. Yao, ShinyGO: A graphical gene-set enrichment tool for animals and plants. *Bioinformatics* **36**, 2628–2629 (2020).
14. A. Khan, A. Mathelier, Intervene: A tool for intersection and visualization of multiple gene or genomic region sets. *BMC Bioinformatics* **18**, 1–8 (2017).
15. B. Chen, *et al.*, Regulation network of colorectal-cancer-specific enhancers in the progression of colorectal cancer. *Int. J. Mol. Sci.* **22**, 1–13 (2021).
16. I. Dunham, *et al.*, An integrated encyclopedia of DNA elements in the human genome. *Nature* **489**, 57–74 (2012).
17. S. Noguchi, *et al.*, FANTOM5 CAGE profiles of human and mouse samples. *Sci. Data* **4** (2017).
18. A. M. Bolger, M. Lohse, B. Usadel, Trimmomatic: A flexible trimmer for Illumina sequence data. *Bioinformatics* **30**, 2114–2120 (2014).
19. H. Li, R. Durbin, Fast and accurate short read alignment with Burrows-Wheeler transform. *Bioinformatics* **25**, 1754–1760 (2009).
20. D. Kim, J. M. Paggi, C. Park, C. Bennett, S. L. Salzberg, Graph-based genome alignment and genotyping with HISAT2 and HISAT-genotype. *Nat. Biotechnol.* **37**, 907–915 (2019).
21. P. Danecek, *et al.*, Twelve years of SAMtools and BCFtools. *Gigascience* **10** (2021).
22. T. Kouno, *et al.*, C1 CAGE detects transcription start sites and enhancer activity at single-cell resolution. *Nat. Commun.* **10**, 3 (2019).

23. A. R. R. Forrest, *et al.*, A promoter-level mammalian expression atlas. *Nature* **507**, 462–470 (2014).

24. R. Andersson, *et al.*, An atlas of active enhancers across human cell types and tissues. *Nature* **507**, 455–461 (2014).

25. K. R. Sanson, *et al.*, Optimized libraries for CRISPR-Cas9 genetic screens with multiple modalities. *Nat. Commun.* **9**, 1–15 (2018).

26. K. Labun, *et al.*, CHOPCHOP v3: Expanding the CRISPR web toolbox beyond genome editing. *Nucleic Acids Res.* **47**, W171–W174 (2019).

27. G. Teo, *et al.*, SAINTexpress: Improvements and additional features in Significance Analysis of INTeractome software. *J. Proteomics* **100**, 37–43 (2014).

28. H. Choi, *et al.*, SAINT: Probabilistic scoring of affinity purificationg-mass spectrometry data. *Nat. Methods* **8**, 70–73 (2011).

29. G. Kim, *et al.*, Easy and accurate protein structure prediction using ColabFold. *Res. Sq.* (2023). https://doi.org/10.21203/rs.3.pex-2490/v1.

30. J. Jumper, *et al.*, Highly accurate protein structure prediction with AlphaFold. *Nature* **596**, 583–589 (2021).

31. M. Mirdita, *et al.*, ColabFold: making protein folding accessible to all. *Nat. Methods* **19**, 679–682 (2022).

32. R. Evans, *et al.*, Protein complex prediction with AlphaFold-Multimer. *bioRxiv* 2021.10.04.463034 (2022). https://doi.org/10.1101/2021.10.04.463034.

33. G. Kim, *et al.*, Easy and accurate protein structure prediction using ColabFold. *Res. Sq.* (2023). https://doi.org/10.21203/rs.3.pex-2490/v1.

34. J. Abramson, *et al.*, Accurate structure prediction of biomolecular interactions with AlphaFold 3. *Nature* **630**, 493–500 (2024).

35. M. Coll, J. G. Seidman, C. W. Müller, Structure of the DNA-bound T-box domain of human TBX3, a transcription factor responsible for ulnar-mammary syndrome. *Structure* **10**, 343–356 (2002).

36. D. Sehnal, *et al.*, Mol∗Viewer: Modern web app for 3D visualization and analysis of large biomolecular structures. *Nucleic Acids Res.* **49**, W431–W437 (2021).

37. N. Doumpas, *et al.*, TCF/LEF dependent and independent transcriptional regulation of Wnt/β-catenin target genes. *EMBO J.* **38**, e98873 (2019).

38. D. Zimmerli, *et al.*, TBX3 acts as tissue-specific component of the Wnt/β-catenin enhanceosome. *Elife* 1–17 (2020). https://doi.org/10.1101/2020.04.22.053561.

39. G. Pizzolato, L. Moparthi, S. Söderholm, C. Cantù, S. Koch, The oncogenic transcription factor FOXQ1 is a differential regulator of Wnt target genes. *J. Cell Sci.* **135** (2022).

40. Y. Perez-Riverol, *et al.*, The PRIDE database resources in 2022: A hub for mass spectrometry-based proteomics evidences. *Nucleic Acids Res.* **50**, D543–D552 (2022).
